# Riboswitch-inspired toehold riboregulators for gene regulation in *Escherichia coli*

**DOI:** 10.1093/nar/gkac275

**Published:** 2022-04-21

**Authors:** Tianhe Wang, Friedrich C Simmel

**Affiliations:** Physics of Synthetic Biological Systems – E14, Physics Department and ZNN, Technische Universität München, Am Coulombwall 4a, 85748 Garching, Germany; Physics of Synthetic Biological Systems – E14, Physics Department and ZNN, Technische Universität München, Am Coulombwall 4a, 85748 Garching, Germany

## Abstract

Regulatory RNA molecules have been widely investigated as components for synthetic gene circuits, complementing the use of protein-based transcription factors. Among the potential advantages of RNA-based gene regulators are their comparatively simple design, sequence-programmability, orthogonality, and their relatively low metabolic burden. In this work, we developed a set of riboswitch-inspired riboregulators in *Escherichia coli* that combine the concept of toehold-mediated strand displacement (TMSD) with the switching principles of naturally occurring transcriptional and translational riboswitches. Specifically, for translational activation and repression, we sequestered anti-anti-RBS or anti-RBS sequences, respectively, inside the loop of a stable hairpin domain, which is equipped with a single-stranded toehold region at its 5′ end and is followed by regulated sequences on its 3′ side. A trigger RNA binding to the toehold region can invade the hairpin, inducing a structural rearrangement that results in translational activation or deactivation. We also demonstrate that TMSD can be applied in the context of transcriptional regulation by switching RNA secondary structure involved in Rho-dependent termination. Our designs expand the repertoire of available synthetic riboregulators by a set of RNA switches with no sequence limitation, which should prove useful for the development of robust genetic sensors and circuits.

## INTRODUCTION

RNA-based gene regulation mechanisms play an important role in many biological contexts such as in the control of metabolic (1,2) and developmental processes (3,4), or in immune response (5,6), and they are found both among prokaryotes and eukaryotes, and even in viruses (7). Mechanisms such as *CRISPR* and *RNAi* are based on protein complexes that require the participation of non-coding RNA molecules (cr/tracrRNA (8) or siRNA/miRNA (9), respectively) to interfere with gene expression at the transcriptional or translational level.

Utilized mainly in bacteria, riboswitches are cis-acting regulatory RNA elements that involve complex folded RNA domains in the 5′-untranslated regions (UTR) of mRNA molecules, which can change their conformation in response to the presence of small metabolites (10). Most riboswitches contain an aptamer region as the recognition domain for the metabolite, which is followed by an expression platform that controls gene expression either by terminating transcription prematurely or by sequestering the ribosome binding site (RBS) sequence to inhibit translation initiation. When small metabolites such as guanine (11,12), adenine (13), or vitamin B12 (14) bind to the aptamer region, they trigger a rearrangement of the aptamer structure, which allosterically induces a conformational change in the expression platform, thus altering the expression of the downstream mRNA sequence (Supplementary Figure S1a) (15).

Inspired by such small-molecule dependent RNA regulators, over the past decade a range of synthetic RNA-based regulatory systems have been developed. Such regulators have already been applied in metabolic pathway engineering (16), in the construction of synthetic gene circuits (17), for the development of biosensors (18,19), in vivo sensors (20,21) and regulators for inducible gene expression (22,23). RNA based gene regulation provides several advantages, which make it particularly interesting for such synthetic applications. Due to the inherent sequence programmability of RNA secondary structural elements, RNA switches can be rationally designed and optimized for orthogonality (19,24), which is further supported by the availability of a wide range of computational tools for sequence design (25,26) prediction of thermodynamic properties and even molecular dynamics (27,28). Compared to protein-based regulators, RNA regulators also represent a relatively low metabolic burden for the host organism (29,30), which is advantageous when scaling to larger systems.

Among the recently engineered synthetic riboregulators, ‘toehold switch riboregulators’ (or briefly ‘toehold switches’) employ a toehold-mediated strand displacement process (31), which had previously been developed in the context of dynamic DNA nanotechnology (32,33). Toehold-mediated strand displacement (TMSD) utilizes the presence of a short, single-stranded DNA or RNA sequence (the ‘toehold’) to enable efficient initiation of a strand displacement process, in which an ‘invader’ strand (carrying a sequence complementary to the toehold) displaces an ‘incumbent’ strand from a duplex (34–36). In the context of toehold switch riboregulators, a 14 nt long toehold domain is attached at the 5′ end of an RNA hairpin that sequesters the RBS of an mRNA molecule. A trigger RNA, which is sequence-complementary to the toehold and stem sequence, can invade the hairpin structure by TMSD, resulting in the release of the RBS and thus activation of translation (31,37).

Compared to a different approach towards RNA-dependent riboregulators developed earlier (38), utilization of the toehold strategy allowed to increase the dynamic range of the switches by more than an order of magnitude and also enabled the development of large sets of mutually orthogonal regulators (Supplementary Figure S1b). This, in turn, enabled the development of multi-input logic gates based on toehold switches (39). Apart from translational activation, TMSD recently was also successfully applied to the development of translational repressors (40). Recently, prediction of toehold-switch performance was also shown to be amenable to a deep learning methodology (41).

In an alternative approach, Chappell et al. developed RNA transcriptional activators (termed small transcription activating RNAs, or STARs), which exploit anti-sense RNAs to regulate the formation of an intrinsic transcriptional terminator located upstream of a reporter gene through a strand invasion process (42). Both approaches - toehold switches and STARS - can be combined to create heterogeneous RNA regulators that exert control over gene expression both at the transcriptional and translational level, and thus potentially increase the overall dynamic range of the regulation process (43).

In contrast to most naturally occurring riboswitches, toehold switches and STARS take RNA molecules as their input, and are thus ‘sequence-programmable’. This property has been used for the development of in vitro biosensors for the detection of viral nucleic acids (37,44), single-nucleotide variations (45), and other analytes (46). Toehold switches could even be shown to respond to the expression of the endogenous sRNA RyhB in E.coli (31). Apart from the detection of nucleic acids, RNA-responsive elements naturally lend themselves as components for genetic circuits (39), as they can be ‘wired up’ rationally based on their nucleotide sequences, and accordingly a large variety of sequence-orthogonal elements can be designed (31,39).

Current designs for synthetic, RNA-triggered riboregulators still exhibit certain shortcomings such as leaky translation in the OFF state and sequence constraints for the trigger RNAs, respectively. These issues compromise the dynamic range and orthogonality of these components, which poses a challenge for the further development of genetic circuitry composed of larger numbers of RNA regulators, and also their potential use as sensors of endogenous RNA molecules. In the present work, we therefore sought to further expand the repertoire of rationally designed toehold regulators whose RNA inputs can be freely chosen. We applied the TMSD principle to a variety of mechanisms of translational and transcriptional regulation, which were inspired by the architecture of naturally occurring riboswitches, and thus combined functional elements of synthetic and natural gene regulators.

The overall design of our riboregulators is characterized by a toehold hairpin structure at the 5′UTR, which includes an anti-sense or anti-anti-sense sequence within the loop that is complementary to a functional sequence domain further downstream, followed by an expression platform. Binding of a trigger RNA at the toehold induces an allosteric rearrangement of the switch domain via TMSD, which either leads to premature termination of transcription or to sequestration of the RBS and hence repression of translation initiation (Supplementary Figure S1c). The switching process is thus similar to that found in riboswitches, but it is induced by an RNA input rather than by a small molecule. We find that our design strategy indeed provides a viable approach to reduce the sequence constraints on the trigger RNA molecules, as the sequence for the toehold hairpin structure responsible for the initial allosteric rearrangement can be chosen freely.

Next to translational activators and repressors, we realized transcriptional activators based on interference with the formation of an intrinsic terminator. Of note, we were also able to demonstrate RNA regulators that utilize a Rho-dependent mechanism, which also allowed the realization of a transcriptional repressor. Combination of translational and transcriptional control on a single transcript enabled the implementation of a genetic NOR gate with an overall enhanced ON/OFF ratio. At present, however, operation of our switches required the relatively high RNA levels provided by transcription with T7 RNA polymerase, limiting their application to synthetic gene circuitry.

## MATERIALS AND METHODS

### RNA structure design

We designed the riboswitch-inspired toehold riboregulators by combining a modified toehold switch structure (31) with rationally designed regulatory sequences. Initially, we adopted sequences for the toehold and stem region (30 nt in total), which had previously shown excellent *in vivo* performance (with ON/OFF ratios of 665 and 557, respectively, and proven input orthogonality) (31). We then modified the original toehold hairpin structure by removing the uppermost base-pairs in the stem (which were not opened through TMSD in the original design) to increase the refolding efficiency. We then put a sequence domain targeting one of the regulatory downstream sequence elements (i.e. RBS, t22 terminator, or rut) into the loop region and adjusted this sequence to prevent stacking within the loop region. The loop sequences were typically chosen to be partially complementary to the targeted sequence and to additional 2–3 nt on their 5′ sides. These sequence domains were included to act as internal toeholds to increase refolding efficiency. We calculated the free energy of each structural domain of the riboregulator to guarantee the thermodynamic favorability of a secondary rearrangement induced by TMSD, including the free energy of toehold hairpin (ΔG_toehold_hairpin_), anti-hairpin (ΔG_anti-hairpin_), and anti-anti-hairpin (ΔG_anti-anti-hairpin_). Sequences for the hairpin were designed to follow the order: ΔG_toehold_hairpin_ < ΔG_anti-anti-hairpin_ < ΔG_anti-hairpin_ and ΔG_toehold_hairpin_ < ΔG_anti-hairpin_. All RNA structures were designed and simulated using NUPACK (39) and RNAfold from ViennaRNA Web Services (40). All ΔG terms were calculated using NUPACK at 37°C with the default parameter set (Serra and Turner, 1995). We adjusted the length of the stem of toehold hairpin (12 nt- 16 nt) and the free toehold region (14 nt-18 nt) to maintain the stability of the toehold hairpin.

### Plasmid construction

All DNA oligonucleotides were purchased from Eurofins Genomics Germany. Each toehold switch hairpin was PCR amplified from the template annealed from DNA oligos (Supplementary Data - Primer list). Toehold riboregulators and triggers were both controlled by a T7 RNA polymerase promoter. Sequences for the trigger RNA molecules were coded on the same plasmids as the toehold riboregulators. All plasmids were constructed using the 3A assembly (47) method and blunt-end cloning (48) (Supplementary Materials). Purification of all PCR products was performed using a Monarch® PCR & DNA Cleanup Kit (NEB). Recombinant plasmids were purified using a mini-prep kit (QIAprep® Spin Miniprep Kit, QIAGEN).

### Bacterial cell culture

Recombinant plasmids were transformed into chemically and electrically competent *E. coli* cells:

Turbo NEB (glnV44 thi-1 Δ(lac-proAB) galE15 galK16 R (zgb-210::Tn10)TetS endA1 fhuA2 Δ(mcrB-hsdSM)5, (rK–mK–) F′[traD36 proAB + lacIq lacZΔM15]) were used for cloning using a standard protocol, while BL21 DE3 (F–ompT gal dcm lon hsdSB(rB–mB–) rne131) were used for gene expression. Experiments were performed for three biological replicates collected over separate days. All cloning strains (Turbo NEB) were grown in LB medium (Carl Roth), while the expression strains (BL21 DE3, NEB) were grown in 5 mL M9 minimal medium (5 × M9 minimal salts, 1 mM thiamine hydrochloride, 0.2% Casein hydrolyzate, 2 mM MgSO_4_, 0.1 mM CaCl_2_, 20 mM glucose) in conical tubes (50mL), with appropriate antibiotics at 37°C for different time durations. (See also Supplementary Data - Experimental procedures).

### Total RNA extraction

Total RNA for quantitative PCR (qPCR) experiments was extracted from *E. coli* by using a TRIzol reagent (Invitrogen™) RNA isolation protocol. For each biological replicate, a single colony was picked from an LB agar plate of an overnight transformation and precultured in 500 μl of M9 medium containing the appropriate antibiotics for several hours until the OD_600_ reached a value of 0.5. Volumes of 10 μl each of the precultured cells were added to Conical Tubes containing 4985 μl (1:500 dilution) of antibiotic containing M9 medium and grown for 4 to 5 h under the same incubation condition until the OD_600_ reached the value 0.5. Then to each culture 5 μl of 1M IPTG was added (1:1,000 dilution) to induce the expression of T7 RNA polymerase, followed by further incubation for 3h. After culture, cells were collected from a 1.5 mL volume by centrifugation at 1,000 g. for 5 min. After discarding the supernatant, the remaining cell pellet was suspended in 1 mL of TRIzol reagent and homogenized by pipetting, followed by incubation at room temperature for 5 min. Cell debris was removed by centrifugation at 12,000 g for 2 min. Samples were then transferred into new 2 mL tubes, 200 μl chloroform (Carl Roth) was added and the samples were mixed for 20 s and incubated at room temperature for 3 min. After incubation, the samples were centrifuged for 10 min at 12,000 g at 4°C, and 400 μl of the aqueous layer containing the RNA was transferred into a fresh 1.5 mL tube. 500 μl isopropanol (Carl Roth) were added to the aqueous phase, the sample was inverted and incubated at room temperature for 10 min and then centrifuged for 10 min at 14,000 rpm at 4°C. After centrifugation, the supernatant was discarded, the remaining pellets were properly washed in 1 mL of pre-cooled 70% ethanol (Carl Roth) and centrifuged for 2 min at 14,000 rpm at 4°C. The remaining supernatant was discarded and the samples were dried at room temperature. 40 μl of RNase-free ddH_2_O were added to resuspend the pellets for further digestion.

### DNase treatment of total RNA extracts

Purified total RNA samples were treated with DNase I (NEB) with reaction buffer for 1h to remove the remaining plasmid and genome DNA. After digestion of the DNA, 0.5 M EDTA solution (Invitrogen™) were added to samples (1:100 dilution) to prevent Mg^2+^ dependent RNA hydrolysis. DNase I was denatured by heating at 75°C for 10 minutes. The RNA samples were further purified using an RNA-clean up kit (NEB). The concentration and quality of the purified total RNA samples were quantified via the 260/280 and 260/230 ratios using a Nanodrop 8000 spectrophotometer (Thermo Fisher).

### Normalization of total RNA, reverse transcription and qPCR measurements

The concentrations of the purified samples were determined with the Nanodrop 8000 spectrophotometer, and then diluted to 500 ng/μl of total RNA in 10 μl RNase-free ddH2O. 1 μl of diluted total RNA, 0.5 μl of 10 μM reverse transcription primer (Supplementary Table S1), 2 μl of 10 mM of dNTPs (New England BioLabs) 5X Reverse Transcriptase Buffer (biotech rabbit) and RNase-free ddH_2_O (up to 18.5 μl) were incubated for 5 min at 65°C and cooled on ice for 5 min. 1 μl of RevertUP II reverse transcriptase (biotech rabbit), 0.5 μl of Murine RNAse inhibitor (NEB) were then added, and the solution was incubated at 55°C for 1 h, 80°C for 5 min and then stored at − 20°C. qPCR was performed using 5 μl of Lunar qPCR master mix (NEB), 1 μl of cDNA and 0.5 μl of 0.5 μM mCherry qPCR primers (Supplementary Data - Primer list) and up to 10 μl RNase-free ddH_2_O (dilute cDNA if necessary). A iQ™ 5 real-time PCR machine (BIO-RAD) was used for data collection using the following PCR program: 95°C for 2 min, followed by 30 cycles of 95°C for 15 s and 60°C for 35 s. All of the measurements were followed by melting curve analysis. Strips of 8 Thermo-Tubes in White & Clear Caps (Thermo Fisher) were used for all measurements. Results were analyzed using iQ™ 5 software (BIO-RAD). To quantify the relative abundance of cDNA concentration, a 5-point standard curve covering a 10,000-fold range of quantified linear DNA concentrations was measured and used to determine the relative mCherry cDNA abundance in each sample (Supplementary Data - Figure S12). Non-template controls were run in parallel to each measurement to check contamination and nonspecific amplification or primer dimers. Additionally, qPCR was performed on total RNA samples to confirm that no DNA plasmid was detected under the same conditions. Melting curves were recorded to confirm that only a single product was amplified.

### In vitro characterization in a cell-free expression system

All cell-free gene expression reactions were performed using an *in vitro* protein synthesis kit (PURExpress®, NEB) using the standard protocol. In experiments, 10 nM plasmid DNA (final concentration) was expressed in triplicate. 25 μl of each cell-free reaction mixture was transferred to a 384-well plate (BRAND®), covered with a plate seal (Microseal®, BIO-RAD) and placed on a CLARIOstar® plate reader. The temperature was controlled at 37°C, and mCherry fluorescence was measured (EX: 570 nm, EM: 630 nm) every 5 min for 5h.

### Absorbance and fluorescence measurements and analysis

We transformed recombinant plasmids containing toehold riboregulators into *E. coli* electro competent cell (BL21 DE3, NEB) using a standard protocol. *E. coli* cells were cultured on LB agar plates containing 100 μg/ml antibiotics at 37°C overnight. Three colonies were picked and cultured in M9 medium (500 μl) for 4h, followed by a subculture in 5 ml M9 medium in a centrifuge tube (50 ml). Cells were then cultured in a shaking incubator at 37°C for 4–5h until the absorbance (OD_600_) reached a value of 0.5. Subsequently, 1mM IPTG wwas added to the cell culture to induce the expression of T7 RNA polymerase. After overnight culture, 250μl of cell culture was transferred to a 96-well plate (IBIDI 96-well square black) and their fluorescence and OD_600_ was measured using a microplate reader (CLARIOstar®, BMG LABTECH) with the following settings: Excitation/Emission wavelength: 570–20/630–40 nm; Gain value: 1000; focus height: 2.4 mm.

Data were analyzed using MARS data analysis software (BMG LABTECH). OD_600_ and fluorescence values for each replicate were first corrected by subtracting the values of a blank measurement with pure culture medium. The ratio of the absorbance-normalized fluorescence intensities (Fluorescence/OD_600_) was then calculated for the replicates. We calculated mean relative fluorescence intensities values from the replicates, error bars given in the figures represent the standard deviation (s.d.). The ON/OFF ratio (for activation or repression) for each toehold riboregulator was calculated by dividing the relative fluorescence intensities of the corresponding ON and OFF states. A Welch's t-test was calculated to determine statistical significance (*P* < 0.05 or 0.01) between different experimental conditions. Flow cytometry measurements were performed using a BD FACSMelody^TM^ instrument. Cells were diluted (1: 500) into phosphate buffered saline (PBS) and then sampled from the cuvette. Error levels for the fluorescence measurements for cells in the ON and OFF states were calculated from the standard deviation of three biological replicates. Data were analysed using FlowJo 10.1r1 software (FLOWJO) (cf. Supplementary Methods - Fluorescence and absorbance measurements with microplate reader and Flow cytometry, and Supplementary Figure S13).

## RESULTS

### Riboswitch inspired control of translation initiation

Studies of the secondary structure of naturally occurring translational riboswitches have consistently shown (11,49,50) that in the absence of ligands, the RBS of the expression platform is either completely free or completely sequestered by an anti-RBS sequence within a duplex, thus allowing or precluding binding of the ribosome, respectively. In the absence of its ligand, the aptamer module of a riboswitch masks the corresponding anti-RBS or anti-anti-RBS sequence, resulting either in an ‘OFF’ or an ‘ON’ riboswitch. In an OFF switch, binding of a ligand to the aptamer induces a refolding process that releases the anti-RBS, enabling interactions with the complementary RBS and thus switching the expression platform into the OFF state. In a ON switch, ligand binding releases the anti-anti-RBS, which in turn sequesters the anti-RBS and thus makes the RBS available for ribosome binding. The utilization of anti-sense RBS sequences in natural riboswitches contributes to the relatively low leak expression of the controlled genes, which usually play an important role in cellular metabolism.

For our synthetic riboregulators, we replaced the riboswitch aptamer domain by a toehold hairpin whose switching via TMSD induces refolding of the expression platform. The toehold hairpin contains an unpaired *cis*-acting regulatory sequence (either an anti-RBS or anti-anti-RBS), which avoids any sequence constraints for the RNA trigger input.

**Figure 1.**
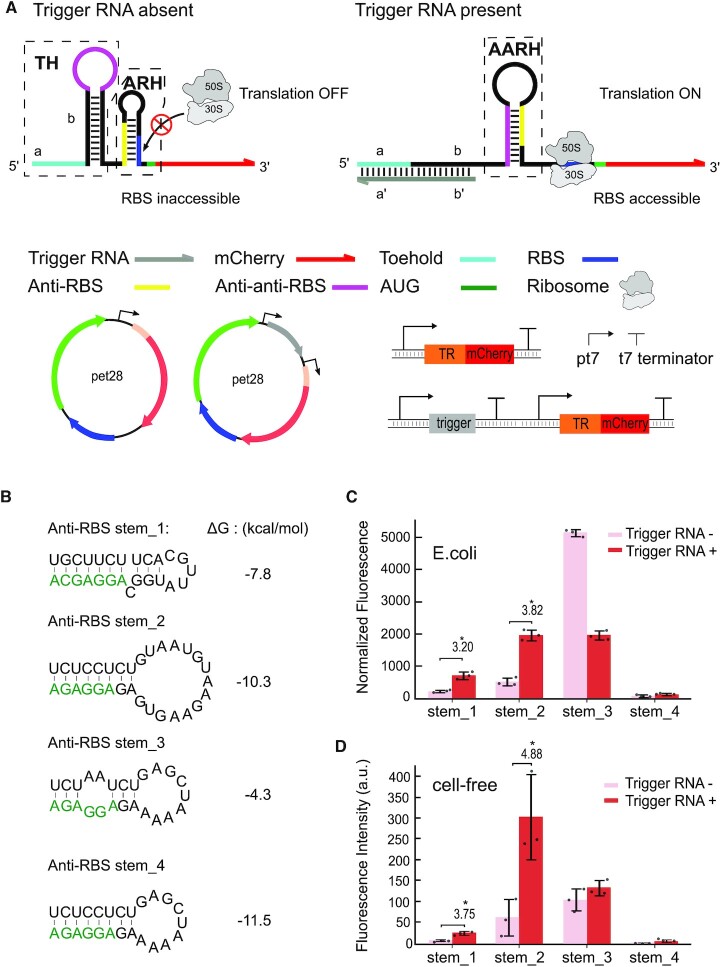
Design and characterization of riboswitch-inspired toehold-riboregulator controlling translational activation. (**A**) Scheme of a toehold riboregulator that activates translation initiation in response to trigger RNA input. In the absence of a trigger RNA (grey), the toehold hairpin (TH) confines an anti-anti-RBS sequence (purple) within its loop region. The RBS (blue) is sequestered within an anti-RBS hairpin (ARH) by an anti-RBS sequence (yellow), which prevents binding of the ribosome. Trigger RNA can initiate a TMSD process at the toehold (light blue), which releases the anti-anti-RBS sequence. The RBS sequestration hairpin is unfolded by the released anti-anti-RBS sequence and forms an anti-anti-RBS hairpin (AARH), which in turn exposes the RBS to the ribosome and allows translation of the mCherry readout (red) to proceed. (**B**) Predicted secondary structure and total free energy of each anti-RBS hairpin - the RBS sequence is highlighted in green. (**C**) Relative fluorescence intensities from *in vivo* measurements in the ON and OFF state for each anti-RBS hairpin, respectively. (**D**) Relative fluorescence intensities obtained in cell-free experiments with riboregulators in the ON and OFF state, respectively. For both the relative fluorescence/OD and fluorescence intensity data, Welch's t-tests were performed for each construct; *P < 0.05, indicating conditions where the fluorescence/OD and fluorescence intensity for the trigger RNA + condition is statistically significantly different from that of the trigger RNA-condition. Error bars represent the standard deviation (s.d.) for biologically independent samples.

**Figure 2.**
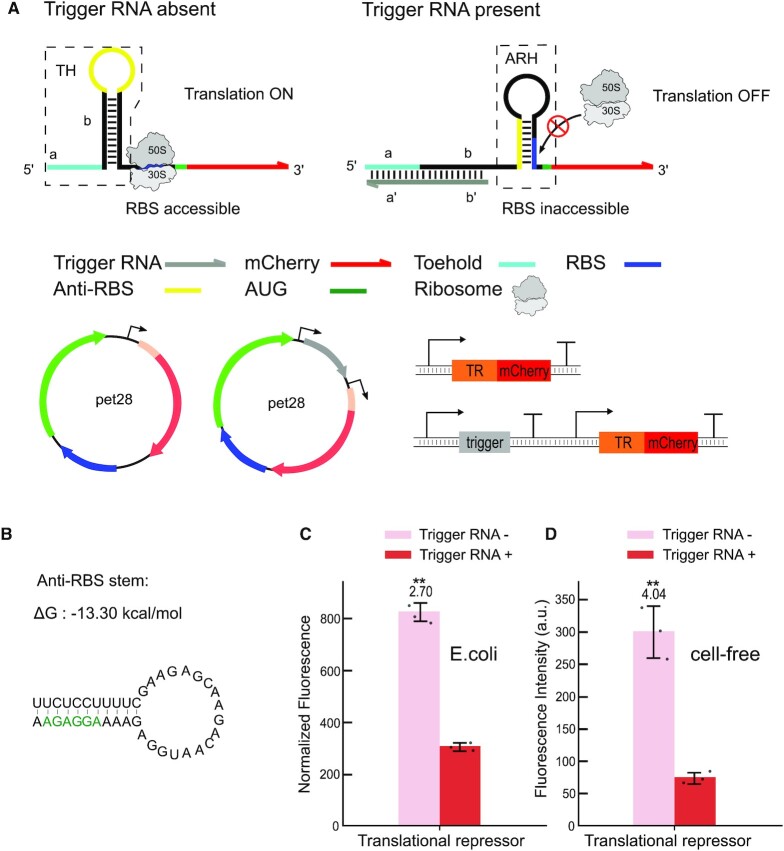
Design and characterization of a riboswitch-inspired toehold-riboregulator controlling translational repression. (**A**) In the absence of a trigger RNA (grey), the toehold hairpin (TH) constrains an anti-RBS sequence (yellow) within its loop region, the RBS is freely accessible and translational initiation is enabled. In the presence of trigger, toehold (light blue)-mediated invasion of the hairpin stem releases the anti-RBS, which leads to formation of anti-RBS hairpin (ARH) and sequestration of the RBS and thus translational repression. (**B**) Predicted secondary structure and total free energy of the anti-RBS hairpin. The RBS sequence is highlighted in green. (**C**) Relative fluorescence intensities in the ON and OFF state of the translational toehold repressor measured *in vivo*. (**D**) *in vitro* relative fluorescence intensities of the translational toehold repressor in its two states measured in a cell-free expression system. For both relative fluorescence/OD and fluorescence intensity data, Welch's t-tests were performed on each construct; *P < 0.05 and **P < 0.01 indicate conditions where the fluorescence/OD and fluorescence intensity for the Trigger RNA + condition is statistically significantly different from that of the trigger RNA- condition. Error bars in c, d represent the s.d. from at least three biologically independent samples.

Our designs for translational activators (ON switches) and repressors (OFF switches) are shown in Figures 1 and 2, respectively (cf. Supplementary Figure S2a & b). The translational activators consist of a 5′ toehold hairpin with an anti-anti-RBS sequence in the loop, followed by different anti-RBS hairpin (AARH) loops. Our AARH design termed anti-RBS stem_1 is derived from the *E. coli thiM* riboswitch (51), which sequesters the RBS in the OFF state of the switch. We varied the ‘natural’ design to include different loop sizes and stem structures (anti-RBS stem_2–4). In each case, activating trigger RNA molecules can bind to the 14 nt long toehold and break up the first hairpin via TMSD. The anti-anti-RBS sequence exposed by this process binds to the anti-RBS sequence, releasing the RBS and thus facilitating translation initiation of the downstream mCherry reporter. A short unpaired region was added between toehold hairpin and anti-RBS hairpin to support the refolding of the anti-anti-RBS hairpin.

Thermodynamically, the TMSD-driven refolding process of our toehold riboregulators is driven by the free energy difference (ΔΔGs) between the conformations attained in the ON and OFF states. In order to ensure switchability of our RNA constructs, we adjusted the folding free energies of the RNA structural motifs present in the absence and presence of a trigger RNA molecule. For example, translational activators in the OFF state (in the absence of a trigger RNA) include a toehold hairpin (ΔG_TH_ = −22.30 kcal/mol) and an anti-RBS hairpin (ΔG_ARH_ = −7.80 kcal/mol). The sum of the free energies of these motifs has to be lower than the alternatively folded anti-anti-RBS hairpin (ΔG_AARH_ = −15.00 kcal/mol) (Supplementary Figure S2 a). Binding of trigger RNA binding followed by TMSD disrupts the toehold hairpin to form a stable double stranded region (ΔG_ds_ = −47.38 kcal/mol < ΔG_TH_ + ΔG_ARH_), which prompts the formation of the anti-anti-RBS hairpin.

For consistency, we designed the toehold hairpins of all of our riboregulators with the same loop size (15 nt) to ensure similar behavior during the initial TMSD process. By contrast, the stem lengths and loop sizes of the anti-RBS, anti-terminator and anti-rut sequence domains depend on the downstream target RNA sequences and were adjusted individually to meet the design rules and ensure an overall ΔΔG < 0.

In order to assess the *in vivo* performance of the translational activator, its components were cloned into a recombinant plasmid and studied in experiments with *E. coli* BL21 DE3 in M9 medium. Both trigger RNA and toehold riboregulators were put under the control of T7 RNA polymerase. In order to activate the riboregulators, IPTG (1mM) was added to an *E. coli* cell culture, which resulted in expression of T7 RNA polymerase (RNAP) by the E.coli BL21 DE3 bacteria. In fact, we found that transcription by T7 RNAP was crucial for the performance of the switches. Experiments with a weaker E.coli promoter led to worse performance or even loss of function (cf. Supplementary Figure S16 and Discussion).

Upon activation, the activator derived from the *thiM* riboswitch displayed a relatively low leak and an ON/OFF ratio of ≈ 3.2, while our best-performing design (anti-RBS stem_2) had a higher leak, but an ON/OFF ratio of ≈ 3.8 (Figure 1c) (Supplementary Materials - Experimental procedures). Interestingly, anti-RBS stem_3 containing two mismatches in the stem shows a very strong leak and reduced activity in the presence of trigger rather than activation, while the most stable AARH anti-RBS stem_4 had a very low leak, but could not be activated. In addition, we also tested our translational activator (anti-RBS stem_1) in LB medium and the performance (ON/OFF ratio ≈ 3.5, Supplementary Figure S17) is similar as in minimal media.

We also tested our translational riboregulators in an *E. coli*-derived cell-free expression system (PURExpress®) to compare their performance with the *in vivo* results. To this end, linear DNA templates containing toehold riboregulators and triggers were added to the cell-free system, and fluorescence intensities were measured with a microplate reader (Methods & Supplementary Methods - *In vitro* protein expression in cell-free system). The *in vitro* results were in line with the *in vivo* experiments, but tended to show a slightly better performance in terms of ON/OFF ratios. The best performing AARH (anti_RBS stem_2) exhibited an ON/OFF ratio of ≈ 4.9 (Figure 1d).

We wish to note that the measured ON/OFF ratios are highly dependent on the experimental details. When assessed using the same experimental workflow, the performance of our translational activators is comparable to that of other previously developed riboregulators (31) (cf. Discussion).

In the case of our translational repressors, we utilized an anti-RBS to bind the RBS after refolding of the switch with the aim to prevent undesired ribosome invasion in the OFF state and thus improve translational repression. In the ‘ON state’ of the repressor (Figure 2a), the anti-RBS sequence is initially located in the loop of the toehold hairpin, leaving the RBS freely accessible for ribosome binding and thus allowing translation of the mCherry reporter. Upon binding of the trigger RNA and strand invasion into the toehold hairpin, the anti-RBS sequence is released, followed by sequestration of the RBS (Figure 2b) and thus repression of translation initiation of the mCherry reporter. To ensure proper switching, the free energy of the toehold hairpin (ΔG_TH_ = −23.10 kcal/mol) was designed to be lower than the alternatively folded anti-RBS hairpin (ΔG_AR_ = −15.90 kcal/mol). In the translational ON state, i.e. in the absence of trigger, the anti-RBS sequence is thus safely sequestered within the loop of the toehold hairpin (Supplementary Figure S2b). Corresponding in *vivo* experiments for the translational repressor resulted in ON/OFF ratios of ≈ 2.7 (Figure 2c, while *in vitro* experiments with the repressor resulted in a slightly better ON/OFF ratio of ≈ 4 (Figure 2d).

#### Intrinsic terminator dependent transcriptional termination

**Figure 3.**
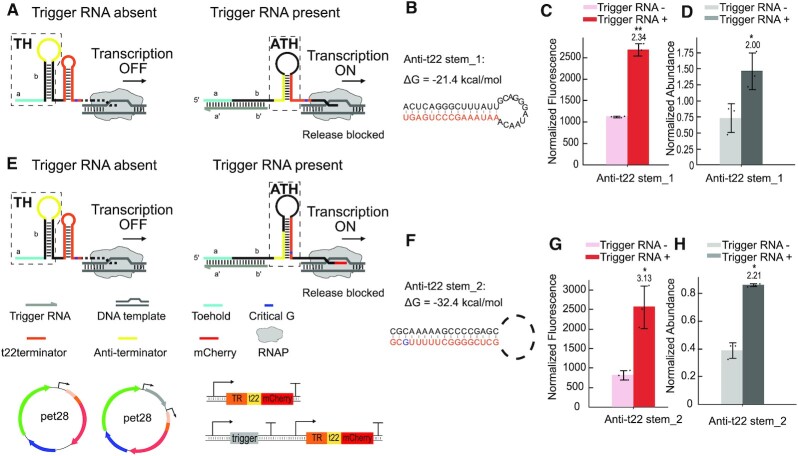
Design and characterization of riboswitch-inspired toehold riboregulator mechanisms controlling transcriptional activation via an intrinsic terminator. (**A**) Anti-t22 stem 1 design: In the absence of a trigger RNA (grey), a transcriptional anti-terminator (yellow) is constrained within the loop of the toehold hairpin (TH), and thus transcription is terminated by the following intrinsic *t22* terminator (orange). In the presence of trigger RNA, trigger binding and TMSD opens the TH, and the released anti-terminator sequence sequesters the terminator in an alternative structure (anti-terminator hairpin, ATH), which allows downstream gene transcription to proceed. (**B**) Predicted secondary structure and total free energy of the anti-*t22* stem, a subsequence of the *t22* terminator is highlighted in orange. (**C**) Relative mCherry fluorescence intensities measured with the transcriptional activator in the OFF and ON state, respectively. (**D**) Normalized abundance of mRNA transcripts characterized by qPCR in the transcriptional OFF and ON state of the anti-t22 stem_1 activator. (**E**) Anti-t22 stem 2 design: In this design TMSD induced refolding of the RNA structure leads to a sequestration of the critical guanine nucleotide (indicated in blue) within the anti-anti-t22 terminator hairpin (ATH) stem, which allows downstream gene transcription to proceed. (**F**) Predicted secondary structure and total free energy of anti-*t22* stem_2, with the sequestered *t22* terminator subsequence highlighted in orange. (**G**) Relative mCherry fluorescence intensities and (**H**) abundance of mRNA transcripts measured by qPCR for the two states of the transcriptional activator. For both fluorescence/OD values and qPCR quantification, Welch's t-tests were performed on each construct; *P < 0.05 and **P < 0.01, indicate that the trigger RNA + condition is statistically significantly different from that of the trigger RNA- case.

A variety of naturally occurring riboswitches are based on the control of transcriptional termination. We therefore sought to apply our approach also to the development of toehold-mediated transcriptional terminators or anti-terminators (Figure 3, Supplementary Figure S3 a & b). The structures of the transcriptional activators (Figure 3a & e) each comprise a toehold hairpin and an intrinsic terminator whose sequence is derived from the late terminator *t22* from phage P22 (47). The *t22* terminator releases an RNA transcript at a critical guanine nucleotide located eight nucleotides away from the terminator stem. Mutating the critical guanine was previously found to result in increased transcriptional readthrough.

In our first design, termination can be interrupted by an anti-terminator sequence, which initially is sequestered within the loop of the toehold hairpin (Figure 3a). When an activating trigger RNA invades the toehold hairpin via TMSD, the anti-*t22* sequence (the sequence on the anti-*t22* stem_1) is exposed in the nascent RNA and can thus hybridize to a 5′ subsequence of the *t22* terminator (Figure 3b) and form an anti-*t22* hairpin (ATH). In our second design, anti-*t22* stem_2 also includes the complementary sequence of the *t22* sequence near its 3′ end, which contains the critical guanine nucleotide of the *t22* terminator (Figure 3f). Binding of trigger RNA results in a refolding of the RNA structure, after which the critical guanine is also sequestered within the stem of the anti-t22 hairpin (Figure 3e). In each case, transcriptional elongation is expected to proceed after refolding.

As shown in Figure 3c and g, *in vivo* experiments with *E. coli* BL21 DE3 carrying plasmids with the components of the two transcriptional activators exhibited ON/OFF ratios of ≈ 2.3 and ≈ 3.1, respectively, indicating that the ‘critical G’ design indeed performs better than the simpler anti-t22 stem_1 design. As an alternative means of estimating the switching efficiency, quantitative PCR (qPCR) was used to characterize the steady-state level of all toehold-mCherry mRNAs in the presence and absence of trigger RNAs in *E. coli* (Figure 3d & e, Supplementary Data - Experimental procedures, Quantitative PCR). In terms of fully transcribed mRNAs, our transcriptional activators showed ON/OFF ratios of 2.0 and 2.2, confirming that control is exerted at the transcription level.

#### Rho-dependent transcriptional termination

Next to transcriptional riboregulators that target intrinsic terminators, we also attempted to control Rho-dependent termination processes. In *E. coli*, about half of the factor-dependent termination processes are Rho-dependent, and they are typically associated with genes involved in metabolism and metabolic control (52–54). Termination factor Rho (55,56) is a homo-hexameric RNA chaperone that binds to nascent RNA by recognizing the Rho utilization sequence (*rut* site) and aborts transcription by pulling the RNA away from the RNAP and DNA template.

A well-studied example of a Rho-dependent termination process is found in the *E. coli* tryptophanase (tna) operon which encodes tryptophanase and permease for tryptophan metabolism (47). At high cellular levels of tryptophan, the ribosome stalls during translation of the tnaC peptide at the *tnaC* stop codon that is adjacent to the *rut* site. The stalled ribosome blocks access of the Rho factor and therefore allows transcriptional elongation to proceed (Supplementary Figure S4). At low tryptophan levels, the ribosome is not stalled, resulting in Rho-dependent termination after completion of tnaC synthesis.

**Figure 4.**
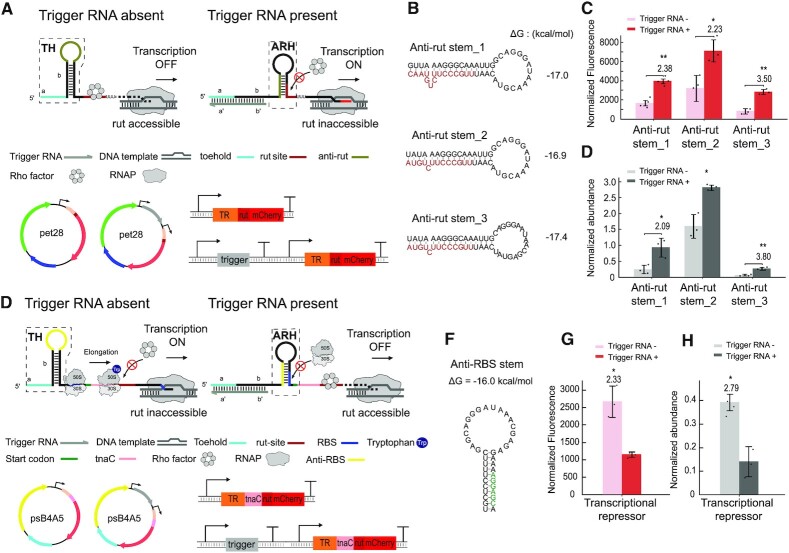
Design and characterization of transcriptional toehold riboregulators based on Rho-dependent termination. (**A**) Principle of a transcriptional activator: In the absence of trigger RNA (grey), the toehold hairpin (TH) confines anti-*rut* sequence (olive) within its loop, while the *rut* site (brown) is exposed to Rho factor, which terminates transcription. Upon invasion of the toehold hairpin (TH) by trigger RNA, the anti-*rut* sequence is released and anti- transcriptional elongation is switched ON. The design of the corresponding plasmids and gene circuits are also shown. (**B**) Predicted secondary structure and free energies of several variants of the anti-*rut* stem, where the *rut* sequence is highlighted in brown. (**C**) Relative fluorescence intensities of the transcriptional activators measured in vivo in the OFF and ON state. (**D**) Corresponding normalized abundance of mRNA transcripts measured by qPCR. (**E**) Scheme of transcriptional repression by a toehold riboregulator, which is based on the *tna* operon. In the absence of trigger RNA (grey), ribosomes can bind to translate the tnaC peptide, followed by stalling at the *rut* site (brown). This prevents Rho from binding and thus allows transcription to proceed. In the presence of trigger, translation of tnaC is disabled and Rho factor can bind to the exposed *rut* site and thus terminate transcription. (**F**) Predicted secondary structure and free energy of the anti-RBS hairpin. The RBS sequence is highlighted in green. (**G**) Relative fluorescence intensities and (**H**) mRNA abundance in the ON and OFF state of the transcriptional repressor and in the presence of 5mM tryptophan. Based on Welch's t-tests, *P < 0.05 and **P < 0.01, indicate conditions where the fluorescence/OD and qPCR quantification for the trigger RNA + condition is statistically significantly different from that of the trigger RNA- condition.

Based on this mechanism, we first attempted to use a toehold riboregulator to activate access of Rho to the *rut* site directly. In the OFF state of this riboregulator, in the absence of trigger RNA an anti-rut sequence is sequestered within the toehold hairpin, making the downstream *rut* sequence accessible for the Rho factor, which leads to transcription termination (Figure 4 a, Supplementary Figure S5). Addition of an activating trigger RNA opens the toehold hairpin and releases the anti-rut sequence, which consequently binds *rut* within the anti-rut hairpin (ARH) and thus prevents Rho-dependent termination.

Rather than utilizing a regulatory sequence for Rho binding, we also designed a toehold transcriptional repressor, in which we controlled the access of the ribosome to the *tnaC* sequence in a similar manner as in the translational riboregulators described above (cf. Figure 2a and Supplementary Figure S6). In the ON state, i.e. in the absence of trigger RNA, an anti-RBS sequence is confined within a toehold hairpin, and therefore the RBS is accessible for ribosome binding and translation of the tnaC peptide encoded on the downstream sequence. In consequence, ribosome stalling during tnaC translation blocks the *rut* site for the Rho factor and thus transcription proceeds (Figure 4e). Binding of an activating trigger RNA followed by TMSD opens the toehold hairpin, enabling sequestration of the RBS sequence by the anti-RBS sequence. As a result, Rho factor binds to the – now free – *rut* site and terminates transcription elongation.

In order to assess the performance of the *rut*-dependent riboregulators, recombinant plasmids coding for the regulators in the presence and absence of trigger RNA were transformed and expressed in *E. coli* BL21 DE3 with additional tryptophan in the medium. Comparison of the resulting mCherry expression end levels resulted in ON/OFF ratios of up to 3.50 for the transcriptional activator (Figure 4c), and up to 2.33 for the transcriptional repressor (Figure 4g). We also used qPCR to quantify the steady-state level of all toehold-mCherry mRNAs in the presence and absence of trigger RNAs in *E. coli* (Figure 4d & h), which were found to be in close agreement with the fluorescence data.

### A logic NOR gate based on combined transcriptional and translational toehold repression

**Figure 5.**
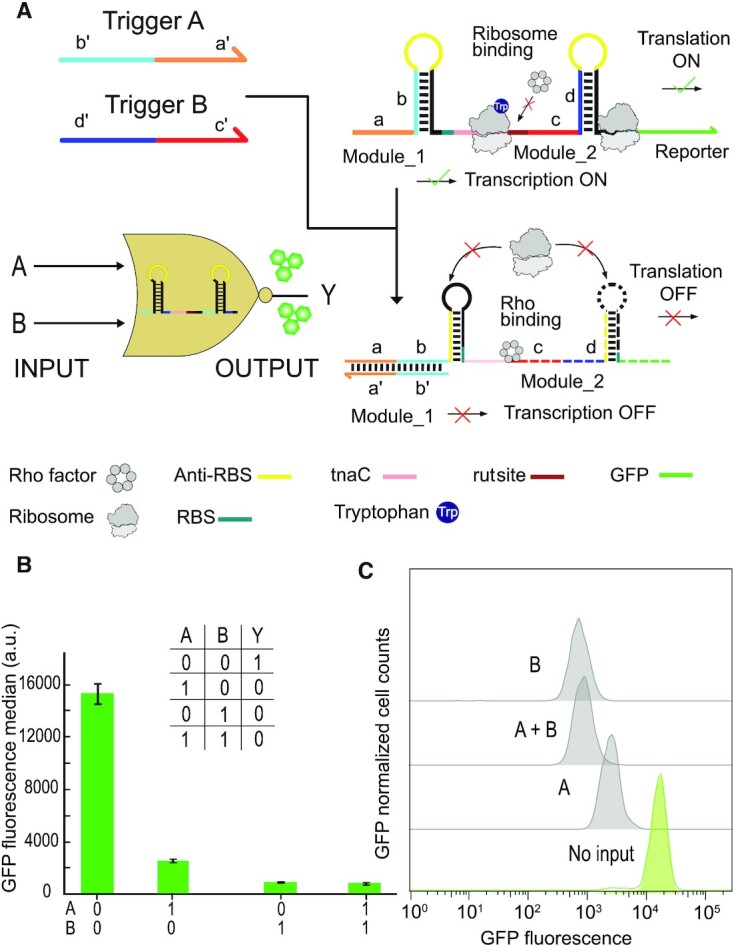
A NOR logic gate based on riboswitch-inspired transcriptional and translational toehold switches. (**A**) The switch is composed of two modules which each control translation from an RBS. The function of the first module is to switch translation of the tnaC peptide, which results in blockage of the rut site by the ribosome (cf. Figure 4). This in turn prevents termination by Rho factor and thus allows transcription to proceed. The second module is a translational toehold repressor for the GFP reporter gene (cf. Figure 2). Binding of the two trigger RNAs lead to transcriptional termination and translational repression, respectively. (**B**) GFP fluorescence output of the NOR gate for different input combinations, measured by flow cytometry in the presence of 5 mM tryptophan (required for the *tna* operon). Given are the mean values from three biologically independent samples, error bars represent their standard deviation. (**C**) GFP fluorescence histograms for the NOR gate toehold riboregulator in the absence and presence of inputs obtained from a single flow cytometry run.

We finally investigated whether it is possible to combine transcriptional and translational control within a single transcript. To this end, we fused the *tna* operon-based transcriptional repressor with a translational repressor, which is expected to generate a logic NOR gate that only results in active gene expression in the absence of the two corresponding trigger RNAs (Figure 5a, Supplementary, Figure S7). Genetic constructs containing the NOR gate were co-transformed into *E. coli* BL21 DE3 with plasmids coding for the different combinations of trigger RNAs, followed by bacterial culture and quantification of the fluorescence output with a microplate reader (Supplementary Figure S7) or via flow cytometry (Figure 5 b & c). The fluorescence data demonstrate NOR gate performance as desired. Notably, the ON/OFF ratio for the ‘transcriptional part’ of the switch is on the order of 7 according to flow cytometry (Figure 5b), whereas the ratio for the translational part or for the combined inputs is > 15 (see Discussion).

In principle, tryptophan could be interpreted as a third input, which controls the transcriptional module of the gate. In the absence of tryptophan, transcription in the absence of trigger A would be reduced by a factor of ≈ 2 compared to the operation with 5 mM tryptophan. Correspondingly, Trp concentration could be used to modulate the maximum output level of the NOR gate.

## DISCUSSION

We designed and characterized a series of riboswitch-inspired riboregulators which are switched by RNA inputs via a toehold-mediated strand displacement (TMSD) process. These riboregulators combine structural features of natural riboswitches, and the switching principle of recently developed synthetic riboregulators. Instead of using a natural, small molecule-responsive RNA aptamer to control the interaction of a cis-regulatory sequence with an expression platform, we placed a regulatory RNA sequence (e.g. an anti-RBS sequence) into the loop region of a toehold hairpin which could be switched by TMSD, inducing refolding of the riboregulator and thus controlling translational initiation or transcriptional termination. Various factors contribute to the observed performance of the translational and transcriptional switches, which relate to the kinetics and thermodynamics of the switches as well as the details of their experimental assessment.

### Quantification of the performance of the riboregulators

In the present work, the performance of the riboregulators was quantified by comparing the endpoint of the fluorescence of bacterial cultures normalized by the density (or OD) in the respective ‘ON’ and ‘OFF’ states of the switches. This enabled us to assess and rank different riboregulator designs with respect to each other. We wish to note, however, that the actual value of the ON/OFF ratio was found highly dependent on the details of the experimental approach. For instance, when investigating previously described toehold riboregulators (31,39) with reported fold changes of more than 600 using our experimental workflow, we obtained ON/OFF ratios of only ≈ 7 (Supplementary Figure S10). We obtained different values for this measure when using RFP or GFP as a fluorescent reporter, or when quantification was performed with Fluorescence/OD, RT-qPCR or via flow cytometry. In the following, we assume that the overall trends observed when varying the design of the riboregulators are robust with respect to the choice of quantification method.

### Influence of promoter choice

We found that our riboregulators performed best when both trigger and riboregulator were transcribed by T7 RNAP. For comparison, we tested two of our riboregulators using a weaker promoter (the *E. coli* promoter BBa_J23119) for transcription of either trigger or riboregulator, or both (Supplementary Figure S16). When riboregulator anti-RBS stem_1 was transcribed from the strong T7 promoter, but trigger RNA was generated from the weaker *E.coli* promoter, we found a strongly reduced ON/OFF ratio for the switch (Supplementary Figure S16a). Transcription of translational activator anti-RBS stem_2 from the *E.coli* promoter led to a strong leak expression even in the absence of trigger, which is in line with results from a previous study on toehold switches (31). In fact, transcription of both riboregulator and trigger led to no switching at all (Supplementary Figure S16b). These findings suggest two distinct effects of the choice of promoter on the performance of the system: First, fast transcription is required to enable fast co-transcriptional folding of the riboregulator and thus prevent binding of ribosomes to the nascent switch (57), which would result in leaky translation. Second, transcription of trigger by T7 RNA polymerase generates higher intracellular concentrations, which are required for switching the riboregulators via toehold-mediated strand displacement (cf. also the discussion of intracellular RNA concentrations and sensing below). With an eightfold higher maximum transcription speed for T7 RNAP than for *E.coli* polymerase (57), and transcription from a high copy plasmid number (pET28b has copy number ≈ 40), trigger and riboregulator concentrations are expected well in the μM range. As overexpression of genes in *E. coli* using T7 RNAP is known to inhibit cellular growth (58,59), we also tested the effect of the generation of trigger RNA by T7 RNAP in *E. coli* BL21 DE3 on bacterial growth. Compared to a negative control, in which only T7 RNAP is expressed, but no T7 promoter is present, we found an increase in doubling time from ≈ 30 to ≈ 60 mins (Supplementary Figure S14).

### Translational activators

Translational activators are supposed to be translationally inactive in the OFF state. The natural strategy to suppress translation found in riboswitches is the use of an anti-RBS/RBS hairpin stem, in which the RBS is sequestered. Our experiments indicate that the hairpin stem has to be stable enough to prevent invasion by the ribosome, but not too stable to prevent switching altogether (Figure 1b & c). Further, sufficiently fast transcription of the riboregulators appears to be required to prevent binding of ribosomes to the nascent RNA (cf. discussion on the use of T7 RNAP above).

We initially designed and characterized several toehold-translational activators whose anti-RBS/RBS sequences were rationally chosen (Anti-RBS stem_2,3,4) and formed hairpin stems of different stability (Figure 1b). Indeed, the most stable hairpin (Anti-RBS stem_4) resulted in the lowest leak, but also did not lead to a strong increase in gene expression in the presence of trigger. The slightly less stable anti-RBS_2 structure performed best in terms of ON/OFF ratio, but with the trade-off of a higher leak expression. We also replaced the *in silico* designed anti-RBS/RBS hairpin by the corresponding structure taken from a natural *thiM* riboswitch from *E. coli* (Anti-RBS stem_1). This naturally occurring anti-RBS hairpin comprises an RBS (aggagc) of lower efficiency compared to that (aggaga) of the other anti-RBS/RBS hairpin stems (RBS calculator (52), Supplementary Table S2), and contains a bulge formed by an unpaired cytosine next to the RBS. Even though the resulting structure had a lower predicted thermodynamic stability than Anti-RBS_2, it had a lower leak *in vivo*, and the second-best ON/OFF ratio obtained for our design variations. The comparatively high leak expression from anti-RBS stem_2 presumably is caused by the large size of the hairpin loop, which potentially allows undesired ribosome binding and translation initiation. Our results thus suggest that ribosome invasion (generating leak) can be prevented by appropriate secondary structure, and potentially is more severe *in vivo* than in the cell-free context. The latter may be the consequence of the ≈ 10-fold lower ribosome concentration in a cell-free system. In agreement with previous findings (60,61), we also found that strategically placed bulges in the toehold stem can improve TMSD efficiency by introducing a forward bias into the strand invasion process (Supplementary Figure S11).

For our riboregulators, we adopted the sequence of the toehold and its adjacent hairpin loop from a previous study on toehold riboregulators (31), which had been tested for input orthogonality with 12 other trigger RNAs with random sequences. In order to confirm the specificity of the design, we tested the performance of translational activator anti-RBS stem_1 with three additional trigger RNAs with scrambled sequences. None of them was found to activate expression appreciably (Supplementary Figure S15).

### Translational repressors

For translational repressors, kinetic considerations become more important than for the activators. In the presence of trigger RNA, translational repressors need to be switched into a translationally inactive state quick enough to avoid leaky translation from an accessible RBS. Transcription of the riboregulator at a speed of ≈ 10–100 nt/s defines a time window of ≈ 1 s for trigger binding to the toehold and disruption of the toehold hairpin (or prevention of its formation). For our repressors, we therefore designed a relatively strong anti-RBS hairpin that is expected to sequester the RBS following toehold-mediated switching of the toehold hairpin (Figure 2b). While our design is shown to work, in principle, we observed relatively strong leakage in the OFF state (in the presence of trigger RNA) (Figure 2c), indicating that TMSD and formation of the anti-RBS hairpin does not occur fast enough to prevent binding of ribosomes to the RBS. Compared to the *in vivo* case, cell-free experiments show a slightly reduced leak in the OFF state of the repressor, which again might be a consequence of the lower ribosome concentration in the cell-free reaction. Due to the reduced leak, the cell-free translational repressor also exhibits a better ON/OFF ratio than *in vivo*.

### Transcriptional riboregulators

The function of transcriptional riboswitches, which are based on termination or anti-termination, critically depends on the kinetics of transcription, ligand binding and refolding of the expression platform (62). Natural riboswitches respond to small metabolites, which have to be present at relatively high concentrations (in the μM to mM range) for switching, and whose binding to the riboswitch's aptamer domain is associated with a relatively low change in free energy. By contrast, our transcriptional riboregulators have to respond to trigger RNAs, which bind with a much higher ΔG, but which are present at typically lower concentrations (in the nM to μM range, see below). A sufficiently high concentration and a correspondingly high on-rate of the triggers is crucial for the functioning of the switches, however.

In a previously developed type of transcriptional regulators termed STARs (42), small trans-acting RNA molecules were utilized that contained an anti-terminator sequence to regulate the formation of the intrinsic terminator and thus control downstream gene transcription. While this strategy results in efficient transcriptional activation, the trigger RNA necessarily includes a part of the complementary sequence of the intrinsic terminator and thus cannot be chosen without constraints. By contrast, our design strategy (Figure 3) leaves the anti-terminator sequence unpaired and confined within the loop of the toehold hairpin, which avoids any sequence limitations for the trigger RNA. An interesting outcome of our experiments is the sensitivity of the performance of the riboregulators to sequence details in the switching domains. For instance, we confirmed that the termination process from the *t22* terminator can be suppressed efficiently, when its ‘critical’ guanine nucleotide is included in the anti-terminator/terminator stem, which is in line with the conclusions of previous work (63,64).

We also demonstrated transcriptional toehold activators and repressors that interfere with Rho-dependent termination (Figure 4), for which we modified the Rho utilization (rut) sequence of the naturally occurring transcriptional switch of the *tna* operon. Experiments with the wildtype *rut* site alone showed substantial transcriptional read-through and a correspondingly leaky expression of the reporter sequence (Supplementary Figure S8). Several factors may contribute to the observed leak. As the Rho factor has multiple cellular functions other than transcriptional termination, its recruitment to the rut site depends on its availability under the given cellular context. Further, secondary structure close to the rut site might reduce its accessibility for Rho. When we removed some of the original sequence context upstream of the *tna* operon's *rut* site, leaky expression was reduced (Supplementary Figure S8). We also found that the termination efficiency of Rho factor was enhanced by insertion of a transcriptional pausing site (U_7_) right after the *rut* site (Supplementary Figures S8 & S9). Based on these insights, we were able to realize TMSD-based anti-rut regulators that controlled rut accessibility and thus Rho-dependent termination with a relatively low leak transcription and ON/OFF ratios of up to 3.5 (Figure 4a-d).

Previous work on Rho factor binding (52) found that most of the riboswitches and sRNAs that modulate Rho-dependent termination are based on ribosome stalling, which is similar to the mechanism found in the *tna* operon (Supplementary Figure S4). Our transcriptional repressors (Figure 4e-h) were thus designed to control ribosome binding (and thus stalling) in the same way as the translational activators discussed above. Several processes have to play together co-transcriptionally to make the switch work: in the transcriptional ON state (in the absence of trigger), ribosomes need to bind to the RBS quick enough to be able to block the rut site before Rho can bind. In the presence of trigger RNA, however, refolding of the toehold switch needs to take place fast enough to prevent undesired binding of ribosomes and thus facilitate binding of Rho to *rut* – our experiments suggest that the kinetic competition between these processes results in an appreciable leak, but still displays a decent ON/OFF ratio of ≈ 2.3.

### Kinetic considerations and potential for sensing of endogenous RNA molecules

An exciting potential application for RNA-triggered riboregulators is the detection of endogenous RNA species such as mRNAs or small regulatory RNAs (sRNAs). However, at this point our designs do not appear to be sensitive enough for such sensing tasks. The range of concentrations expected for endogenous RNAs in *E.coli* lies in the range from 1 nM to ≈ 10 μM. The lower bound corresponds to a single copy of the molecule in the bacterial cell, while the most highly expressed RNAs are the ribosomal RNAs, which are present at concentrations of ≈ 20 μM (65). mRNA concentrations in *E.coli* have been estimated from single molecule FISH experiments, and were found to lie in the range from ≈ 0.1 nM to 100 nM (66), and the total mRNA concentration is ≈ 1.4 μM (65). Similar values (several 10 nM) can be estimated for sRNAs (67). Whether such small concentrations can be sensed, critically depends on several factors: efficient hybridization of the target with the sensor riboregulator, low leak, and sufficient ON/OFF ratio.

In the case of translational ON-switches, we found that a strong anti-RBS hairpin reduces leaky translation, but also diminishes the switchability of the structure. All other designs – the translational repressors and the transcriptional regulators – depend on a kinetic competition, e.g. either between trigger and ribosome binding, or between trigger binding and transcriptional termination (other factors such as the speed of transcription and folding of the secondary structure also play a role (62)).

In the first case, the target molecules would have to be present at similar concentrations or higher than the concentration of free ribosomes, i.e. those not bound to other mRNAs and engaged in translation, which has been estimated to be on the order of 500 nM (68). In the second case, hybridization of the trigger and the nascent mRNA has to take place in the time window between transcription start and formation of the terminator hairpin, which we estimate to be on the order of 1 s (62). With an estimated RNA association rate in *E.coli* of ≈ $3 \times {10^5}{M^{ - 1}}{s^{ - 1}}$ (67), efficient switching would require trigger concentrations above 3 μM. In addition, hybridization rates are strongly dependent on secondary structure and the presence of RNA-binding molecules, which may further reduce the efficiency of the target binding (69).

All these considerations indicate that our current designs only allow detection of RNA species, which are present at relatively high copy numbers, corresponding to concentrations in the μM range. This is consistent with our finding that the riboswitch-inspired riboregulators performed well only when transcribing the triggers and switches from a T7 promoter (see above).

We therefore also sought to improve the efficiency of a transcriptional activator by utilizing the bacterial RNA chaperone Hfq. Binding of Hfq to RNA containing a specific Hfq-binding motif protects the RNA from degradation (increasing the cellular concentration) and promotes RNA hybridization reactions *in vivo* (70). However, modification of trigger RNA with an Hfq-binding hairpin did not result in an appreciable improvement in performance (Supplementary Figure S18). As Hfq is thought to promote rather weak RNA interactions, it is likely to not have an effect in the case of our ‘optimized’, secondary structure-free triggers and toehold hairpins.

### Realization of cellular logic computation

In principle, RNA-based regulatory mechanisms are ideal for the implementation of cellular computing circuits, as the sequence-specificity of RNA interactions allows a rational design and ‘wiring’ of the different components of the circuits. In this respect, our riboswitch-inspired riboregulators have the benefit that the RNA trigger sequences (the ‘input’) can be independently chosen from the more or less fixed sequences required for gene regulation (i.e. RBS/anti-RBS or terminator/anti-terminator sequences).

Input sequences can be chosen independently and orthogonally to trigger their respective toehold hairpins – e.g. the sequences for triggers A and B in the NOR gate demonstrated in Figure 5 had been previously used in the context of toehold switches - they were chosen to be orthogonal and have no biological meaning. As also exemplified by the NOR gate, combining transcriptional and translational regulation within a single switch leads to a comparatively compact design, and also yields an improved ON/OFF ratio compared to a single switch.

However, we also found that combining several switches in the 5′ untranslated region of a single transcript appears to be less modular than naively expected. We attempted to realize a range of other logic gates with this strategy, but in most cases the switches were non-functional or showed poor performance. For instance, an IMPLY gate can be constructed by fusing a translational activator and a translational repressor (Supplementary Figure S19). For this gate, one would expect a low output only in the presence of the trigger for the translational repressor. Due to strong leakage, the IMPLY gate did not show a clear ‘Boolean’ behavior.

Putting several switches in a row appears to be challenging, and our results indicate that the different components do not act independently and thus cannot be combined simply in a modular fashion. Potentially, optimization of such logic gates (or more complex functions) could be achieved with a screening approach combined with machine-learning methodology, as recently demonstrated for toehold switches (41).

## DATA AVAILABILITY

The experimental data sets are either included in this submission, the supplemental information, or are available from the authors upon request. Flow cytometry data have been uploaded to the Zenodo repository under DOI: 10.5281/zenodo.5174952

## Supplementary Material

gkac275_Supplemental_Files
