# Supplementary material for: Riboswitch-inspired toehold riboregulators for gene regulation in Escherichia coli
Source: Nucleic Acids Res. 2022 Apr 21;50(8):4784–98. doi: 10.1093/nar/gkac275 (PMC9071393; doi:10.1093/nar/gkac275)
Supplement: gkac275_Supplemental_Files [file gkac275_supplemental_files.zip › Wang_Riboregulators_SI_rev1_highlighted.pdf]

Supplementary materials

Contents

Experimental procedures ***2***

Culture media **2**

Cell culture **2**

Fluorescence and absorbance measurements with microplate reader and Flow cytometry **3**

***In vitro* protein expression in cell-free system** **4**

Supporting Information ***5***

Design rationale for riboswitch-inspired toehold riboregulators **5**

Anti-RBS translational toehold riboregulators 6

Anti-terminator transcriptional toehold riboregulators 7

The *tna* operon 9

Anti-*rut* toehold riboregulators 10

A toehold riboregulator based on the *tna* operon 11

A NOR gate based on combined transcriptional and translational riboregulation **12**

Additional control experiments **13**

Leak expression under Rho-dependent transcription termination 13

mCherry expression controlled by the original toehold switch 14

Improving TMSD efficiency via mismatches in the toehold hairpin stem 15

qPCR calibration curve 15

Flow cytometry measurements 16

The effect of trigger RNA expression on cell growth 16

Orthogonality test with non-cognate RNA triggers and toehold-translational activator 17

Switching behavior of a translational activator transcribed from a constitutive promoter 18

Addition of an hfq binding site to the trigger RNA 19

A gate implementing IMPLY logic 20

Experimental procedures

Culture media

We used LB medium (Carl Roth) and M9 medium (M9 minimal salts, 5X, Sigma-Aldrich, 1 mM thiamine hydrochloride, 0.2% Casein H, 2 mM $MgSO_4$, 0.1 mM $CaCl_2$, 20 mM glucose) for cell cultures, depending on the purpose. LB medium is utilized to culture the cloning strain (Turbo®), and M9 medium is used to culture the strain used for gene expression regulated by the toehold riboregulators (BL21 DE3®, NEB). The antibiotics carbenicillin (100 µg/ml) and kanamycin (100 µg/ml) were added to the media.

Plasmid construction and cloning process

All DNA oligonucleotides were purchased from Eurofins Genomics, Ebersberg, Germany. All toehold riboregulator sequences were constructed by overlap extension PCR, restriction ligation and blunt-end ligation. Firstly, we used overlap extension PCR to amplify the toehold regulatory sequence. Two DNA oligos that share complementary overhang sequences were then annealed in Phusion® Master Mix (NEB) at an appropriate annealing temperature, calculated with the NEB Tm calculator (https://tmcalculator.neb.com/#!/main). DNA polymerase is then used to extend the 3' ends and fill up the the gaps. The *tna* operator sequence used in some of the switch designs was obtained from the *E. coli* genome using PCR with primers (cf. Primer List). In the second step, we added the forward and reverse primers, including the restriction site (*EcoRI* and *SpeI*) sequence (cf. Primer List) to the PCR mix and amplify the target strand. All the PCR products were purified using the Monarch® PCR & DNA Cleanup Kit (NEB). The concentration and quality of purified DNA templates were quantified via their 260/280 and 260/230 ratios using a Nanodrop 8000 spectrophotometer (Thermo Fisher).
In the next step, toehold regulatory sequences were ligated with a reporter gene using restriction ligation. The reporter gene templates (*gfp* and *mCherry*) were obtained from the iGEM parts registry (http://parts.igem.org/Main_Page). Each reporter template was amplified, and restriction sites (*XbaI*, *PstI*) were added on both ends through primers (cf. Primer List). Cloning vectors (pet28b, psb4A5) plasmids were also digested with *EcoRI* and *PstI* and gel purified to remove the digested strands. Last, all three parts (a ratio of inserts to vector of 1:3 was used) were ligated using T4 ligase (NEB) following the standard protocol. The ligation products were then transformed into chemically competent cells (Turbo®, NEB) with a standard protocol. The cell culture was plated on LB agar plates containing 100 µg/ml carbenicillin and 100 µg/ml kanamycin and incubated overnight at 37 °C. A single colony on the plate was picked and checked using colony PCR. We inoculated the selected colony in 5 mL LB medium and incubated overnight at 37 °C. After overnight culture, cells were collected, and plasmids were purified using miniprep kits (QIAprep Spin Miniprep Kit).
Blunt end ligation was used to fine-tune the anti-stem sequence of the riboregulators. We amplified toehold riboregulator constructs together with the vector using primers that include part of the fine-tuned sequence (cf. Primer List), followed by *in vitro* phosphorylation of the PCR products using T4 Polynucleotide Kinase (NEB) using the standard protocol. Then, the phosphorylated PCR products were ligated using T4 ligase at room temperature for 2h, followed by digestion with *DpnI* (NEB) with a standard protocol to remove the remaining original plasmid DNA. The final products were also transformed into the chemically competent cells (Turbo®, NEB). All plasmids used in this study can be found in the DNA construct sequence list.

Cell culture

Bacterial strains were grown in both media using 5 mL culture each in 50 mL centrifuge tubes at 37°C while shaking at 250 rpm. For cloning strains, a colony was picked up from an LB agar plate and inoculated into LB 5mL medium followed by cell culture overnight using the above growth conditions.
For expression strains, which were used to characterize the expression of the toehold riboregulators, we picked up three colonies each from an LB agar plate, followed by a small-scale preliminary culture in M9 medium (500 µl) for 4 hours. This step helps the bacteria to adapt to the new growth conditions in minimal medium. After preliminary culture, we added 10 µl of the pre-culture into a final volume of 5 mL M9 medium for large-scale culture. The production of T7 RNA polymerase by the cells (which in turn transcribes riboregulators and triggers, cf. Main Text) was induced with 1 mM IPTG (Carl Roth) at 0.4-0.5 OD_{600} after 4 hours of growth. In order to induce the *tna* operator used in some of the riboregulators, additional L-tryptophan (Carl Roth) at a final concentration of 5mM was added to the cell culture also at 0.4-0.5 OD_{600} after 4 hours of growth. Fluorescence measurements on the cell cultures with several replicates were obtained after overnight culture.

Fluorescence and absorbance measurements with microplate reader and Flow cytometry

Recombinant plasmids that encode toehold riboregulators together with reporter genes were transformed into electro-competent cells (BL21 DE3 NEB) using a standard protocol. The cell culture was plated on LB agar plates containing 100 µg/ml carbenicillin and 100 µg/ml kanamycin and incubated overnight at 37 °C. Three colonies were picked up from one plate and pre-cultured in M9 medium, followed by large scale culture in a 5mL centrifuge tube. For each riboregulator, we measured three different samples in total on different days. After overnight culture, cell cultures (250 µl) were transferred to a 96-well plate (IBIDI 96-well square black) and characterized via fluorescence and absorbance (OD_{600}) measurements using a microplate reader (CLARIOstar®, BMG LABTECH) with the following settings (Excitation/Emission wavelength: 570-20/630-40 nm; gain value: 1000; focus height: 2.4 mm). In order to measure bacterial growth curves using the microplate reader, the pre-cultured bacteria were added to a total volume 250 µl of M9 medium, containing 100 µg/ml kanamycin. Then cell cultures were transferred to a sealed 96-well plate (Microseal®, BIORAD) and shake cultured in microplate reader (37 °C, 500 rpm). The absorbance (OD_{600}) of the cell culture was measured every 5min in an overnight experiment.
Data were further analyzed using MARS data analysis software (BMG LABTECH). OD_{600} and fluorescence values for each replicate were first corrected by subtracting the values of a blank measurement obtained with culture medium. The ratio of the relative fluorescence intensities (Fluorescence/OD_{600}) was then calculated for each replicate. The mean relative fluorescence intensities values were calculated from these replicates, error bars shown in the Figures represent the corresponding standard deviation (s.d.). The ON/OFF ratio (for activation or repression) for each toehold riboregulator was calculated by dividing the relative fluorescence intensities obtained from a toehold riboregulator in the presence of trigger RNA (ON state) by the relative fluorescence intensities in the absence of trigger RNA (OFF state). A Welch's t-test was applied to determine the statistical significance ($P < 0.05$ or 0.01) of the results obtained under different conditions.
In order to calculate the bacterial doubling time from the growth curves, we determined the exponential growth phase of the bacteria from a log-plot of the OD against time and fitted an exponential function to the corresponding data:

$$N(t) = N_0 e^{\lambda t} \quad (1)$$

$$t_d = \frac{\ln 2}{\lambda} \quad (2)$$

Here $N(t)$ represents the cell density at time t, N_0 is the initial cell density, λ is the growth rate, and t_d is the doubling time.

Flow cytometry of riboregulator logic gates (Fig. 5 of the main paper) was performed using a BD FACSMelody™ cell sorter. Cells were sampled from overnight culture and diluted by a factor of 500 into phosphate buffered saline (PBS). Each sample containing a logic gate regulator and a combination of RNA inputs was measured using GFP^+ as a fluorescent reporter. The forward scatter signal (FSC) was used for thresholding, and 10,000 individual events were measured in forward scatter, side scatter (SSC) and GFP fluorescence (488 excitation /536 emission) using a flowrate setting of 1.

Flow cytometry data were analyzed using FlowJo 10.1r1 software (FLOWJO). We set a gate to remove background events resulting from PBS buffer only. Specifically, we first plotted the histogram of SSC for each experimental group (toehold logic gate RNA with inputs) and the blank (background events) (Figure S15 a). The *E. coli* population had unimodal distributions in SSC and partially overlapped with the blank.
We set the subset gating threshold by removing the overlapping part from the SSC histogram. The remaining events were used to generate a density plot with FSC-H against SSC-H (Figure.S15 b). We further create a second subset by removing the outlier events, which had a low FSC-H ($<10^2$). Then we applied a new subset gating to analyze the relative fluorescence intensities (Median of the GFP-H) of each experimental group. Figure S15 c shows the GFP fluorescence histogram for *E. coli* cells containing toehold gate RNA and inputs. Error levels for the fluorescence measurements are calculated from the s.d. of measurements from at least three biological replicates.

In vitro protein expression in cell-free system

All *in vitro* gene expression experiments with toehold riboregulators were performed using a commercial *in vitro* protein synthesis kit (PURExpress®, NEB) according to the manufacturer's protocol. Linear transcription templates for toehold riboregulators were first amplified using PCR and purified using Monarch® PCR Cleanup Kit (NEB). The concentration and quality (via their 260/280 and 260/230 ratios) of purified DNA templates were quantified using a Nanodrop 8000 spectrophotometer (Thermo Fisher). The molar concentration of each DNA template was calculated via:

$$\frac{Concentration\ (ng/\mu l)\times 10^6}{Molecular\ weight(g/mol)} = Concentration\ (nM)$$

A final concentration of 10 nM DNA template was used in triplicate. 25 µl of each cell-free reaction mixture was transferred to a black 384-well plate (BRAND®), covered with a plate seal (Microseal®, BIO-RAD) and placed on a CLARIOstar® plate reader with the excitation/emission wavelength set to 570-20/630-40 nm, a gain value of 1000 and focus height 2.4 mm. The temperature was controlled at 37 °C, fluorescence intensity was measured every 5 min for 5h.

Supporting Information

Design rationale for riboswitch-inspired toehold riboregulators

The riboregulators investigated in this study were inspired by the previously developed synthetic toehold switches and naturally occurring riboswitches (Figure S1 a, b). Natural riboswitches comprise of an aptamer on the 5' end, whose binding to a cognate small metabolite induces an allosteric effect. A downstream "expression platform", which includes gene regulatory sequences, is affected by the allosteric rearrangement of the RNA structure, resulting in metabolite-dependent control of gene expression. In contrast to riboswitches, the toehold switches exploit toehold-mediated strand displacement (TMSD) by an RNA trigger molecule to induce structural rearrangement and thus regulate gene expression.

In an effort to combine the functional features of natural riboswitches with those of toehold switches, we utilized a toehold hairpin that contains riboswitch-inspired regulatory elements – anti-RBS, anti-anti-RBS or anti-terminator sequences - within the loop region. Binding of the trigger RNA is thus expected to induce a conformational rearrangement that results in the control of the downstream translational initiation or transcriptional termination or (Fig. S1 c).

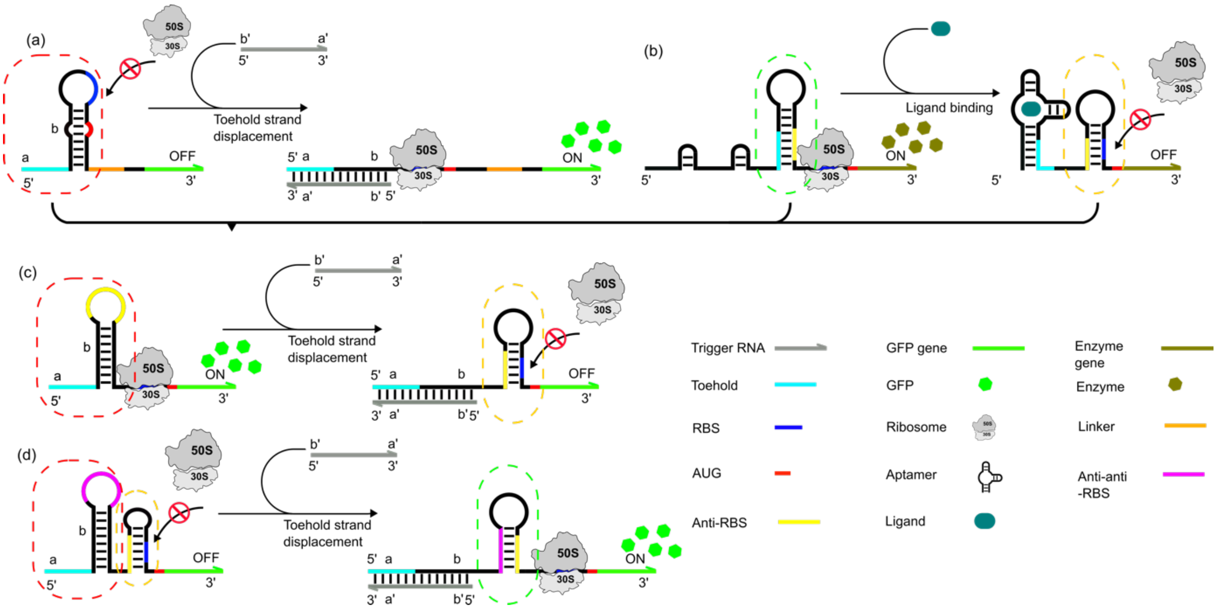

Figure S1. Comparison of the regulatory mechanisms of the toehold switch, riboswitches, and the riboswitch-inspired toehold riboregulators controlling translational initiation and repression investigated in this work. (a) Regulation of translational initiation by a toehold switch. In the absence of trigger RNA (grey), the toehold hairpin (within the red box) includes a free toehold region (light blue) at the 5' end, a loop, and bulge in which the RBS (blue) and start codon (red) are sequestered, preventing the binding of the ribosome to the RBS and inhibiting translational initiation. Trigger RNA binding to the toehold region leads to toehold-mediated strand displacement (TMSD) and unfolding of the toehold hairpin, exposing the RBS and thus enabling translational initiation. (b) Regulatory mechanism of a natural riboswitch controlling translational repression. In the absence of ligands (cyan), the riboswitch contains an aptamer sequence and an anti-RBS hairpin (within the green box) in which the anti-RBS (yellow) sequence is masked, the RBS is accessible and downstream gene translation is enabled. When the aptamer is bound to a ligand, however, the riboswitch adopts a conformation in which the anti-RBS sequence can bind to and block the RBS (within the yellow box), and thus inhibit translation. (c) Schematic representation of a riboswitch-inspired toehold riboregulator controlling translational repression. In the absence of trigger RNA, the riboregulator is in the translational ON state, in which a toehold hairpin (within the red box) sequesters the anti-RBS sequence (yellow) within its loop region. Upon binding of trigger RNA to the toehold region, TMSD results in unfolding of the toehold hairpin, releasing the anti-RBS sequence and thus allowing the formation of the anti-RBS hairpin (within the yellow box), which masks the RBS and inhibits translation. (d) Schematic representation of a riboswitch-inspired toehold riboregulators that controls translational activation. In the absence of trigger RNA, the toehold hairpin (within the red box) confines an anti-anti-RBS sequence (purple) within its loop, which is followed by a hairpin that contains an anti-RBS bound to the RBS in its stem (within the yellow box). Binding of trigger RNA unfolds the toehold hairpin via TMSD, releases the anti-anti-RBS and thus allows formation of the anti-anti-RBS hairpin (within the green box). The RBS is then accessible to the ribosome and translation is initiated.

Anti-RBS translational toehold riboregulators

We first constructed a series of anti-RBS toehold riboregulators, which control the accessibility of the RBS for ribosome binding and translational initiation. We developed two types of translational regulators (Figure S2) which can either activate or repress gene translation through trigger binding and TMSD. The anti-RBS toehold activator is composed of a toehold hairpin and an anti-RBS hairpin (Figure S2a). The toehold hairpin comprises a 14nt unpaired toehold region at the 5' end and a stem-loop, which confines the anti-anti RBS sequence (11 nt) within the loop region. The sequence of the anti-RBS hairpin (ΔG = -7.80 kcal/mol) is derived from the natural *thiM* riboswitch. Trigger RNA binds to the toehold region and initiates TMSD, which unwinds the toehold hairpin and thus releases the anti-anti RBS sequence. The trigger and toehold riboregulator form an intermediate complex, from which the anti-anti-RBS sequence can invade the anti-RBS hairpin. The resulting formation of an anti-anti-RBS hairpin (ΔG = -15.0 kcal/mol) exposes the RBS, resulting in translational initiation. In the case of the anti-RBS toehold repressor, the toehold hairpin comprises a free toehold region (15nt) and an anti-RBS sequence within the loop region (Figure S2b). The RBS is accessible for ribosome binding, allowing the translation process to proceed. Trigger RNA binding and TMSD unwind toehold hairpin and free anti-RBS sequence, leading intermediate state and refolding process. After secondary structure rearrangement, anti-RBS blocks the RBS region, resulting in the formation of anti-RBS hairpins and repression of ribosome binding.

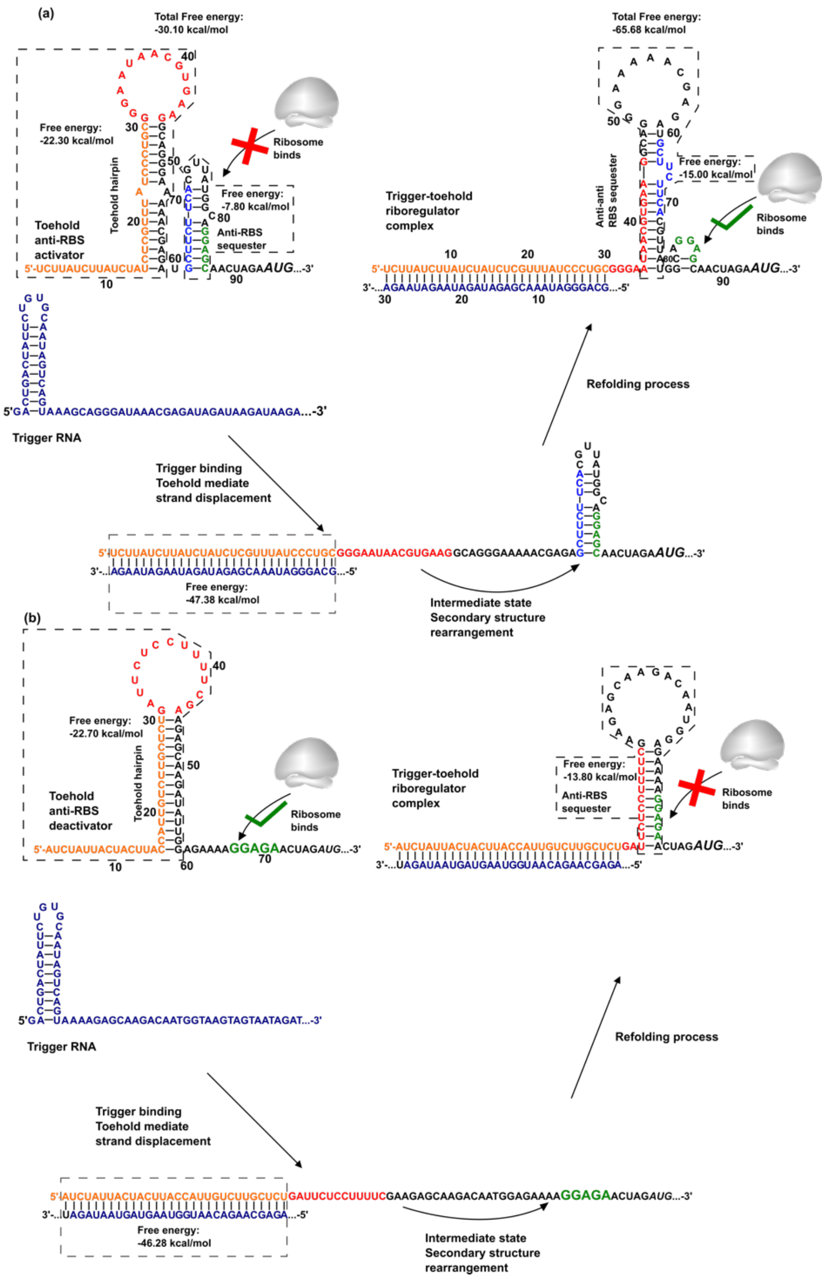

Figure S2. Schematic of translational toehold riboregulators with RNA sequences. a, Regulatory mechanism of a translational activator. The toehold hairpin includes a free toehold region, incumbent sequence (orange), and unpaired anti-anti RBS sequence (red). Anti-RBS hairpin includes an anti-RBS (blue) and RBS (green). Trigger RNA (dark blue) binds to the toehold riboregulator, forming an intermediate complex which promotes refolding of the structure. b, Regulatory mechanism of a toehold translational repressor. The toehold hairpin has a free toehold region, an incumbent sequence (orange), and an unpaired anti-RBS sequence (red). The RBS (green) is unpaired and accessible for ribosome binding. Trigger RNA (dark blue) binds to the toehold riboregulator, which induces refolding of the riboregulator to a translationally inactive state. The trigger RNAs are protected from degradation via a 5' hairpin (shown) and the 3' terminator hairpin (not shown).

Anti-terminator transcriptional toehold riboregulators

The following designs are toehold riboregulators, which control transcriptional termination through an intrinsic (rho-independent) terminator. The toehold transcriptional activators I and II each consist of a toehold hairpin and the *t22* intrinsic terminator (Figure S3a & b). The *t22* terminator hairpin (ΔG = -18.60 kcal/mol) derives from the phage P22 late terminator. The toehold hairpin comprises a 17nt unpaired toehold region at the 5' end and a stem-hairpin which confines the anti-terminator sequence (15 nt) in its loop region. Trigger RNA binds to the toehold region and initiates TMSD, uwinding the toehold hairpin and releasing the anti-terminator sequence. In the resulting intermediate state, the anti-terminator sequence is designed to base-pair either with the releasing site of the *t22* terminator (GCG) (activator I) or with the stem sequence of the terminator (activator II), preventing the release of the nascent RNA and thus allowing transcriptional elongation to proceed.

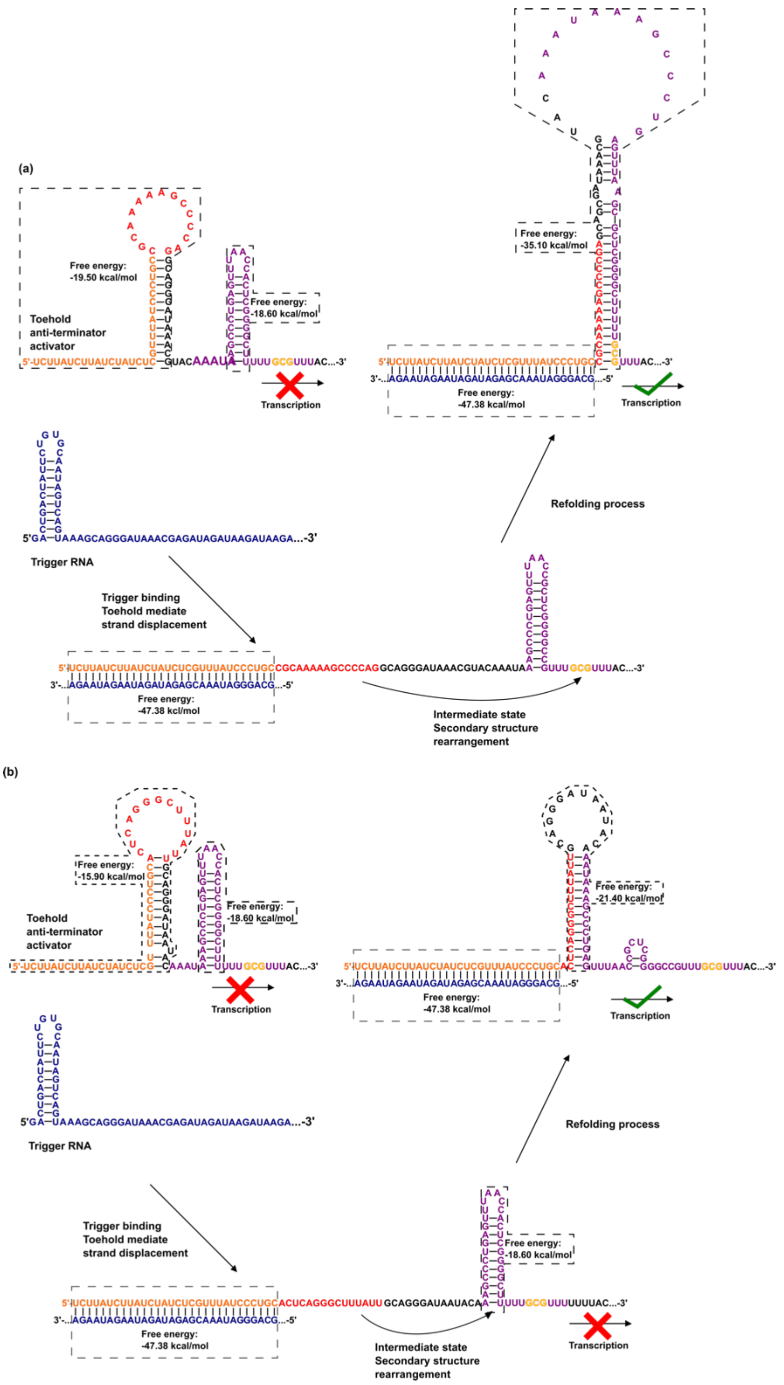

Figure. S3 Schematic representation of transcriptional toehold riboregulators with RNA sequences. a, Mechanism of transcriptional toehold activator I. The toehold hairpin includes a free toehold region, an incumbent sequence (orange), and an unpaired anti-terminator sequence (red). The releasing site (yellow) of the *t22* terminator (purple) is initially unpaired. Trigger RNA (dark blue) binds to the toehold riboregulator, forming a new complex, which includes a double-stranded region and anti-terminator hairpin, in which the releasing site is sequestered. b, Mechanism of transcriptional toehold activator II. In this case the initially sequestered anti-terminator sequence (red) base-pairs with the first half of the stem sequence of the *t22* terminator (purple). As before, binding of trigger results in refolding and thus disruption of the terminator hairpin.

The *tna* operon

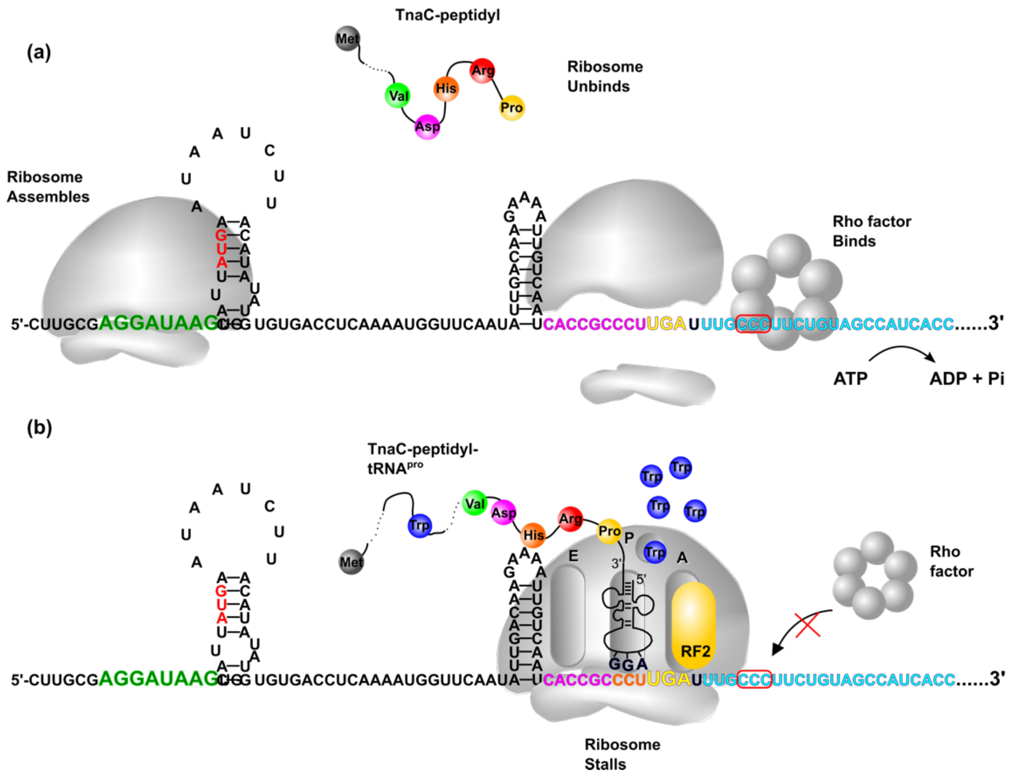

Figure S4. Schematic representation of the mechanism of the *tna* operon in *E. coli* including the *tna* operon mRNA sequence and secondary structures. a, The ribosome initially assembles in the RBS region (in green) and starts translational elongation of the 24-residue leader peptide TnaC (chain of circles) until it reaches the *tnaC* stop codon, UGA (in yellow). At low levels of tryptophan, translation terminates at the *tnaC* stop codon and the ribosome releases, while the Rho factor binds to the *rut* (rho utilization) site (in light blue) and terminates transcriptional elongation. b, High tryptophan levels inhibit the action of release factor RF-2 and thus the TnaC peptide remains covalently linked to tRNA^{pro}. In conseqence the ribosome stalls at the stop codon. The presence of the stalled TnaC-peptidyl-tRNA^{Pro} ribosome complex prevents binding of the Rho factor to the boxA (in purple) and *rut* sites and therefore prevents transcriptional termination.

Anti-*rut* toehold riboregulators

As an alternative to transcriptional toehold switches based on an intrinsic terminator, we designed an anti-*rut* toehold riboregulator (Figure S5), which controls the accessibility of the *rut* site for the binding of the termination factor Rho. Anti-*rut* toehold riboregulators can activated through trigger binding and TMSD.

The anti-*rut* activator is composed of a toehold hairpin and an anti-rut hairpin (Figure S5). The toehold hairpin comprises a 16nt unpaired toehold region at the 5' end and a hairpin that sequesters the anti-*rut* sequence (15 nt) in its loop region. In this state, the *rut* site is accessible for the Rho factor, which binds and leads to transcriptional termination. Trigger RNA can bind to the toehold region, invade the toehold hairpin and thereby release the anti-*rut* sequence. Refolding of the structure sequesters the *rut* site in the stem of an anti-*rut/rut* hairpin (ΔG = -16.90 kcal/mol), allowing transcription elongation to proceed.

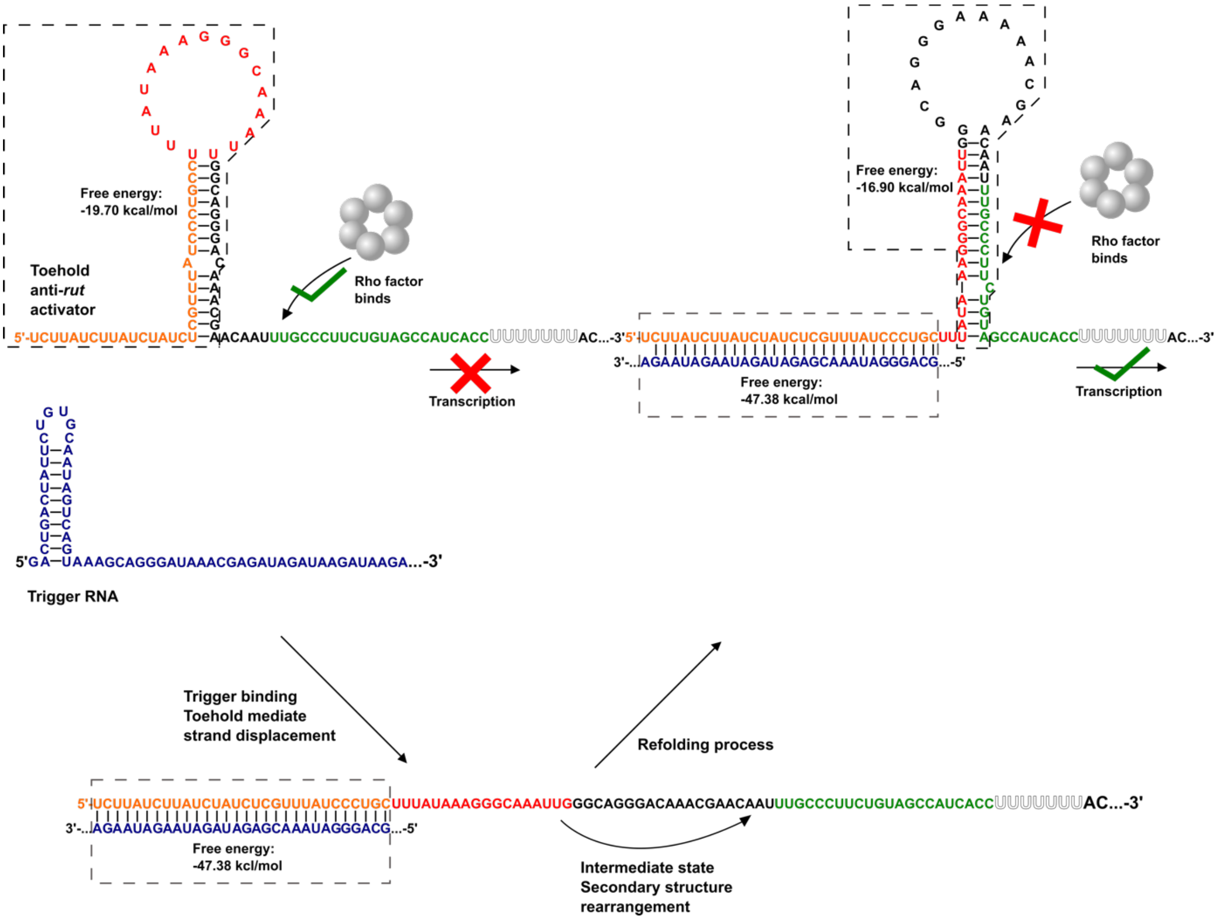

Figure. S5 Schematic of toehold anti-*rut* riboregulators with RNA sequences. a, Regulatory mechanism of a transcriptional toehold activator. An unstructured toehold region precedes the toehold hairpin, which includes an incumbent sequence (orange) and an unpaired anti-rut sequence (red) in the loop region. The rut site (green) for Rho factor binding is accessible, followed by a transcriptional pausing site (U_7). Trigger RNA (dark blue) can bind to the toehold and invade the hairpin. Refolding of the mRNA results in an alternative anti-rut hairpin, which sequesters the rut site.

A toehold riboregulator based on the *tna* operon

Rather than using an anti-*rut* sequence to control the accessibility of the *rut* site, we also utilized ribosome stalling to regulate binding of the Rho factor, which is similar in approach as in the natural *tna* operon (cf. Figure S4). Riboregulators that switch the accessibility of an anti-*tna* sequence can be repressing. Our anti-*tna* toehold repressor (Figure S6) comprises a 15 nt unpaired toehold region at the 5' end and a toehold hairpin that sequesters an anti RBS sequence (15 nt) within its loop region. In the OFF state (in the absence of trigger RNA), the ribosome binds to RNA and stalls at the at the stop codon between boxA sequence *rut* site, allowing transcriptional elongation to proceed. In the presence of trigger RNA, refolding of the riboregulator sequesters the RBS by the anti-RBS and forms a hairpin stem (ΔG ≈ -12.50 kcal/mol), effectively rendering the *rut* site accessible for Rho factor and therefore resulting in transcription termination.

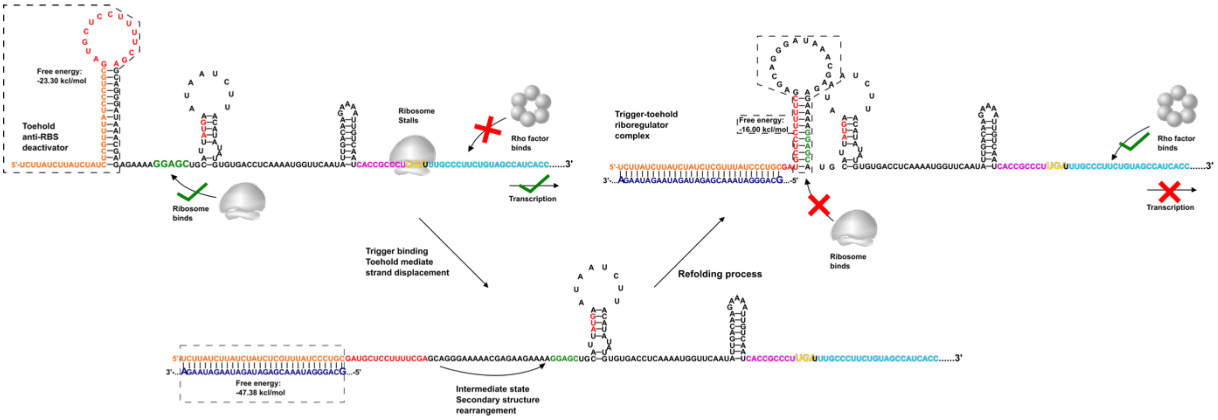

Figure. S6 Schematic of riboregulators derived from the *tna* operon with RNA sequences. Regulatory mechanism of an anti-*tna* toehold repressor. The toehold hairpin is comprised of a free toehold region, an incumbent sequence (orange) and an unpaired anti RBS sequence (red), and the RBS (green) is accessible for ribosome binding. In the OFF state, the ribosome can bind and translate the tnaC peptide starting from the start codon (red AUG) until it reaches the stop codon (yellow) situated between the boxA (purple) and *rut* site (light blue). Translational stalling prevents Rho-dependent termination, allowing transcription elongation. Trigger RNA activates the riboregulator by TMSD into the toehold hairpin. Refolding of the anti-RBS hairpin sequesters RBS from ribosome binding, Rho factor binds to the *rut* site (light blue), which leads to transcriptional termination.

A NOR gate based on combined transcriptional and translational riboregulation

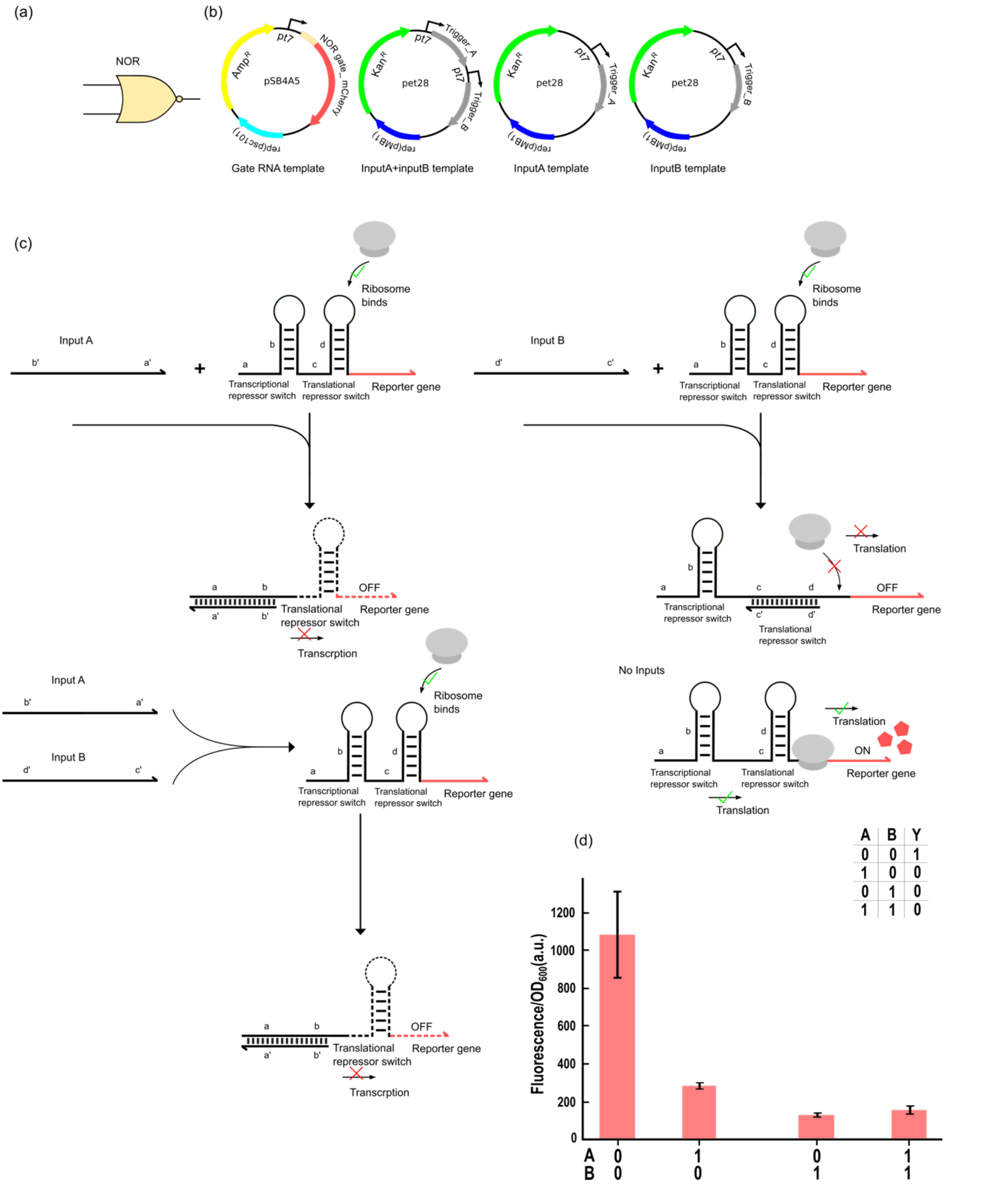

Figure S7. A two-input logic NOR gate device that combines transcriptional and translational repression. a, Logic table for the NOR gate. b, Plasmid schemes. The NOR gate RNA coding for mCherry is under the control of a *pt7* promoter on a pSB4A5 low copy number plasmid. The different trigger RNA combinations are each transcribed from a *pt7* promoter on a pet28 high copy number plasmid. c, The NOR gate RNA consists of a toehold-transcriptional repressor based on the *tna* operon (as in Figure S6) and a translational toehold repressor (as in Figure S2b). In the absence of triggers, ribosomes can bind and translate the reporter gene. In the presence of input A, transcription is terminated prematurely in a Rho-dependent manner as described above. In the presence of input B alone, transcription is not terminated, but translation is repressed. Addition of both triggers also leads to a low output of the reporter protein mCherry. d, Relative fluorescence intensities under ON and OFF state of toehold translational activator respectively. The error bars for the ON and OFF states are from the standard deviation (s.d.) for biologically independent three samples.

Additional control experiments

Leak expression under Rho-dependent transcription termination

We observed leaky expression from the *tna* operator in vivo. First, we tested a wildtype and a modified *tna* operator, which controls the transcription of a downstream mCherry gene (Fig. S8). The wild type *tna* operon includes a sequence coding for the tnaC peptide and the *rut* binding site, followed by a non-coding sequence before the downstream gene. We modified the *tna* operon by replacing the wild-type non-coding sequence with 7 thymidines, which in the RNA transcript mimics the poly-U of the intrinsic terminator to generate an additional transcriptional pause. Both genetic constructs are inserted into a high copy number plasmid and expressed in E. coli BL21 DE3. With the induction of tryptophan in the culture medium, binding of the tryptophan to the ribosome results in ribosome stalling at the stop codon of the tnaC sequence, which blocks the binding of Rho facto to *rut* site and thus allows transcription of the downstream gene.

Next, we removed the tnaC peptide sequence from the operon and retained only the *rut* site to control downstream mCherry gene expression to check the influence of the tnaC peptide sequence. The results still show leaky expression of mCherry under the regulation of *rut* site (Figure S9). However, in the absence of the tnaC peptide sequence, the additional transcriptional pausing site enhanced the Rho-dependent termination compared to the wild-type sequence.

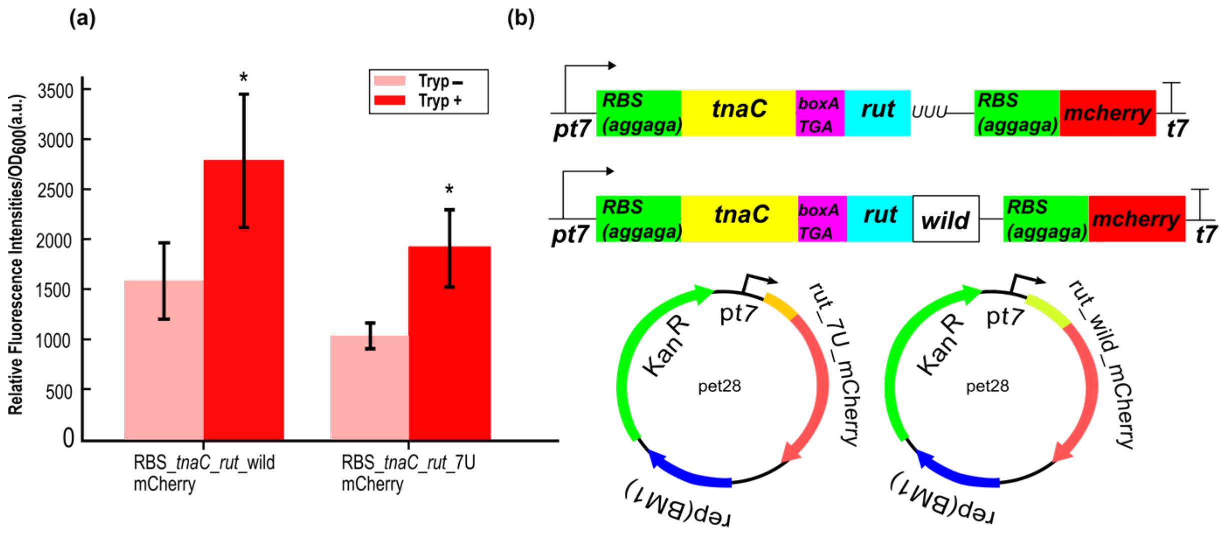

Figure S8. Relative fluorescence intensities measured for mCherry expression under the control of a wild-type and a modified *tna* operator in *E. coli*, BL21 DE3 with and without induction with 5mM tryptophan. T7 RNA polymerase is induced by 1mM IPTG. a, in vivo mCherry expression for the different *tna* operators and induction states. Error bars represent standard deviation over three biological replicates. b, Modified tna operators with *rut*-7U and *rut*-wild. For the modified operator, an additional pausing (7 U) site is inserted right after rut site. Genetic constructs are under the control of a t7 promoter and terminator and coded on a high copy number plasmid pet28.

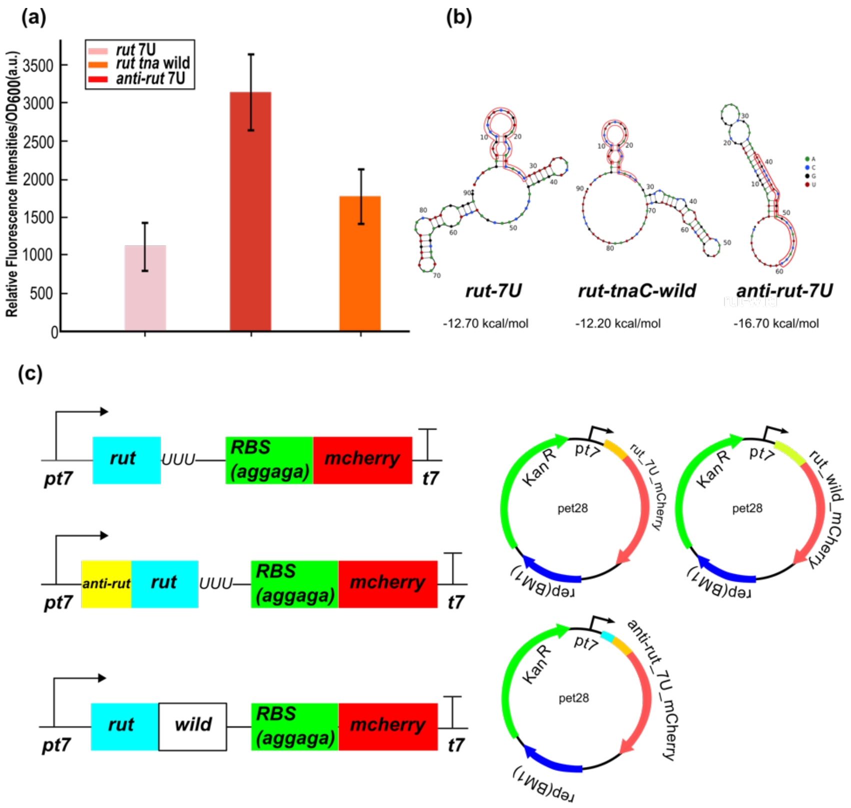

Figure S9. Expression of mCherry in *E. coli*, BL21 DE3 from modified *tna* operators. a, *in vivo* mCherry expression levels for modified *tna* operator rut7U, rut tna wild type and anti-rut 7U. Error bars represent the standard deviation from three biological replicates. b, Prediction of the RNA secondary structures and free energies of the *rut* site including part of the downstream sequence using NUPACK. The *rut* sites of *rut*-7U and rut wild are marked with red frames. C, Scheme of the corresponding genetic constructs. Gene cassettes are under the control of a *t7* promoter and terminator on high copy number plasmid.

mCherry expression controlled by the original toehold switch

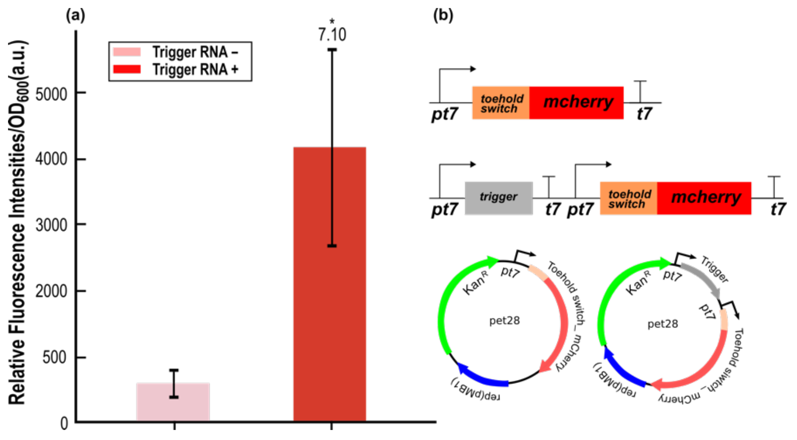

Figure S10. Relative fluorescence intensities of the best performing toehold switch from Green et al. (Ref. 26 of the main paper) controlling the expression of mCherry in *E. coli*, BL21 DE3, where the expression of T7 RNA polymerase is induced with 0.1 mM IPTG. The original toehold switch was reported to have an ON/OFF ratio of 660 in flow cytometer measurements when controlling the expression of GFP. Under our experimental conditions, this ratio is ≈ 7. a, *in vivo* mCherry expression measured as the fluorescence end level/OD_{600} for the OFF and ON state of the toehold switch, i.e., expressed in the absence or the presence of trigger RNA. The ON/OFF ratio of the switch is shown, error bars represent the standard deviation over three biological replicates. b, Genetic constructs for the toehold switch and trigger RNA. Toehold switch-mCherry and trigger RNA are each under the control of a *pt7* promoter and terminator on the same high copy number plasmid and are thus expected to be generated at similarly high expression levels.

Improving TMSD efficiency via mismatches in the toehold hairpin stem

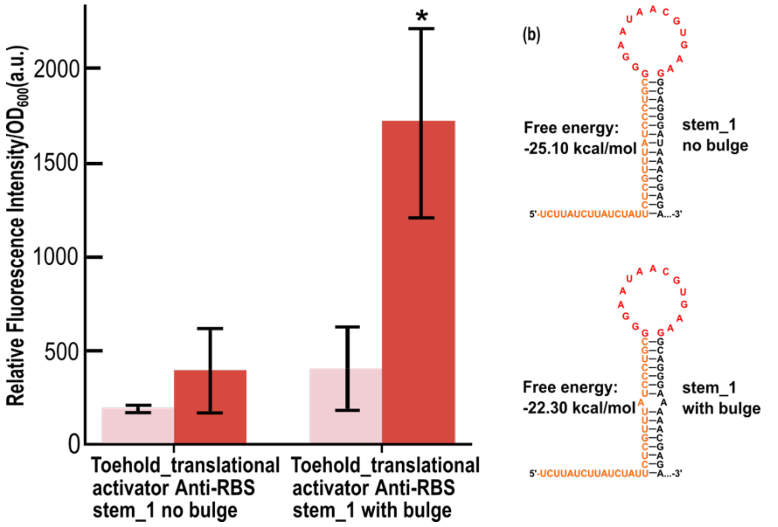

Figure S11. Introduction of mismatches in the toehold hairpin stem improves TMSD. a, *in vivo* mCherry expression levels (fluorescence/OD600 end level) under the control of translational toehold activator stem_1 with and without bulge in the stem, in the absence and presence of trigger RNA. Expression is measured in *E. coli*, BL21 DE3 with induction of the T7 RNA polymerase by 1mM IPTG. Error bars represent the standard deviation over three biological replicates. b, Prediction of mRNA secondary structures and free energies of the toehold hairpins (with and without bulge) using NUPACK.

qPCR calibration curve

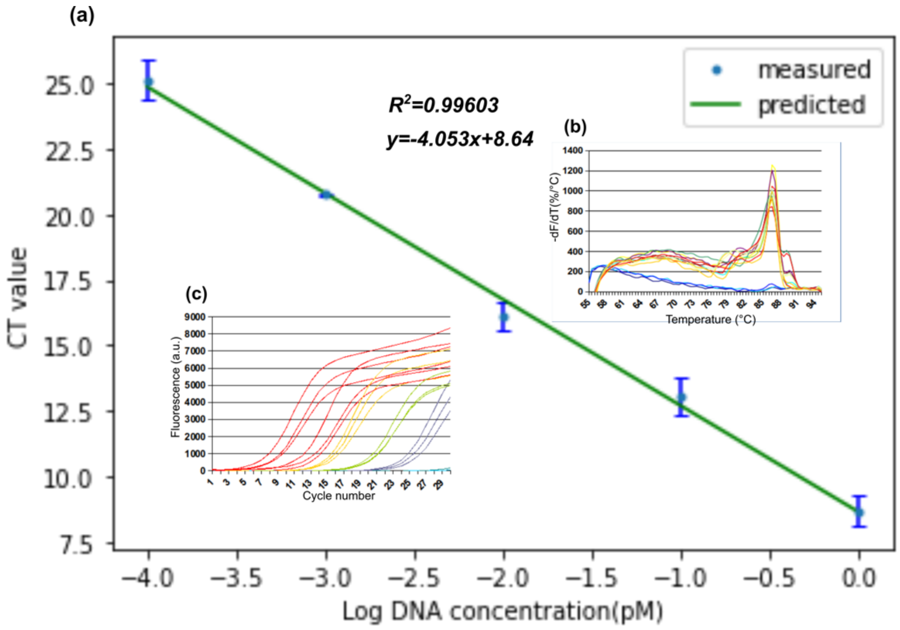

Figure S12. Linearity of the qPCR assay. a, Standard curve of threshold cycle number plotted against the log of the purified mCherry DNA template concentration in picomolar (pM) units. The ten-fold serial dilutions are linear over five orders of magnitude. Data represent three replicates of each dilution. R^2= 0.99603. b, melting curve of dF/dT value of purified mCherry DNA template plotted against the increasing temperature. No significant unspecific amplification was observed in control reactions that did not contain DNA template. c, Amplification plot of the same experiment showing the ten-fold dilution series of DNA concentrations (10^{-4}-10 pM) replicated 3 times.

Flow cytometry measurements

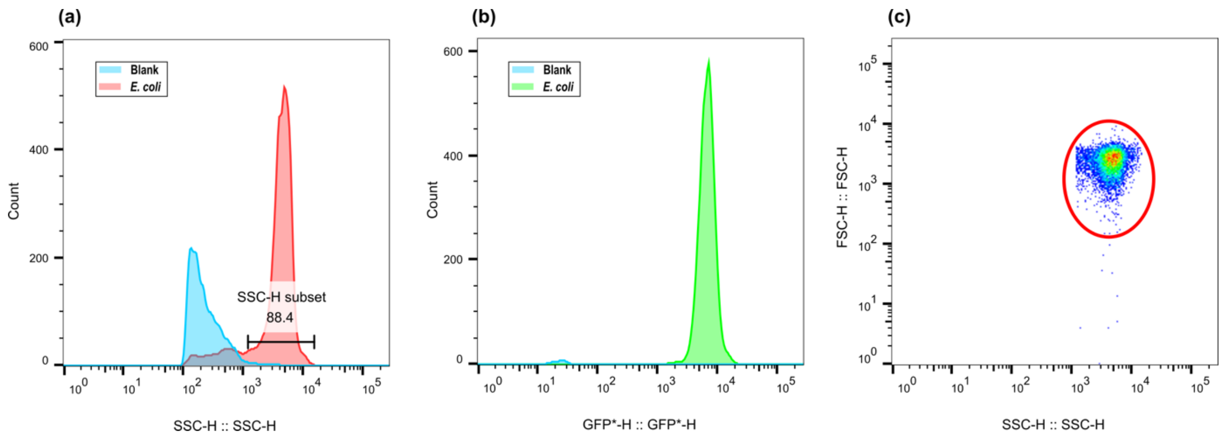

Figure S13. Flow cytometry measurements. a, Histogram of SSC-H for the *E. coli* population and blank. b, Histogram of median of GFP-H for the *E. coli* population and blank. c, SSC-H vs FCS-H density plot. Each dot or point on the plot represents an individual event that passed through the laser of the flow cytometer.

The effect of trigger RNA expression on cell growth

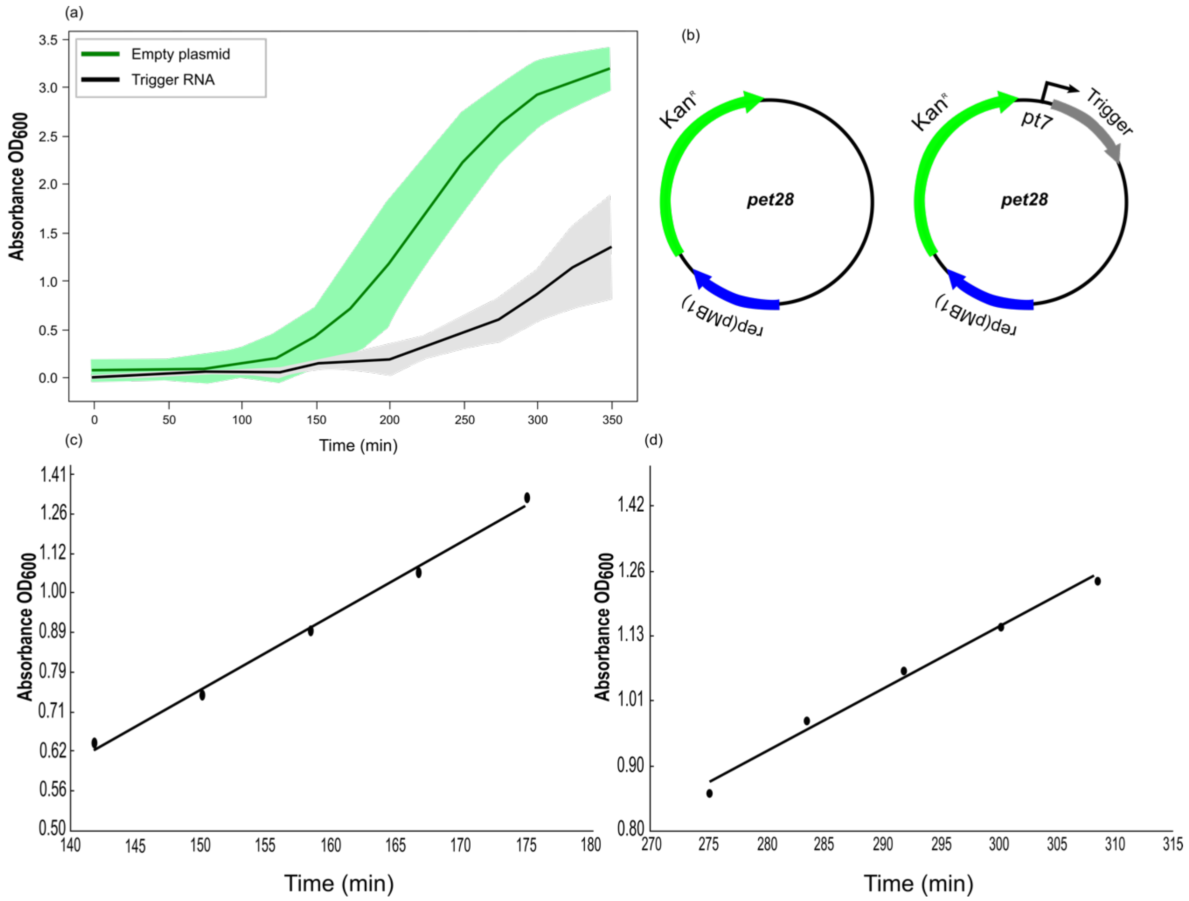

Figure S14. Effects on bacterial growth. a, Growth curves of *E. coli* BL21 DE3 expressing trigger RNA grown at 37°C, 500 rpm for 6 h. The optical densities at 600 nm (OD600) were measured. Shaded error bars represent the average of three replicate experiments. b, Genetic constructs for the trigger RNA. Trigger RNA is under the control of a pt7 promoter and terminator on the high copy number plasmid, an empty plasmid is used for a negative control. c and d, Absorbance in the exponential growth phase of *E. coli* BL21 DE3 that c) carry the empty control plasmid (doubling time ≈ 30 min), and d) that express trigger RNA (doubling time ≈ 65 min).

Orthogonality test with non-cognate RNA triggers and toehold-translational activator

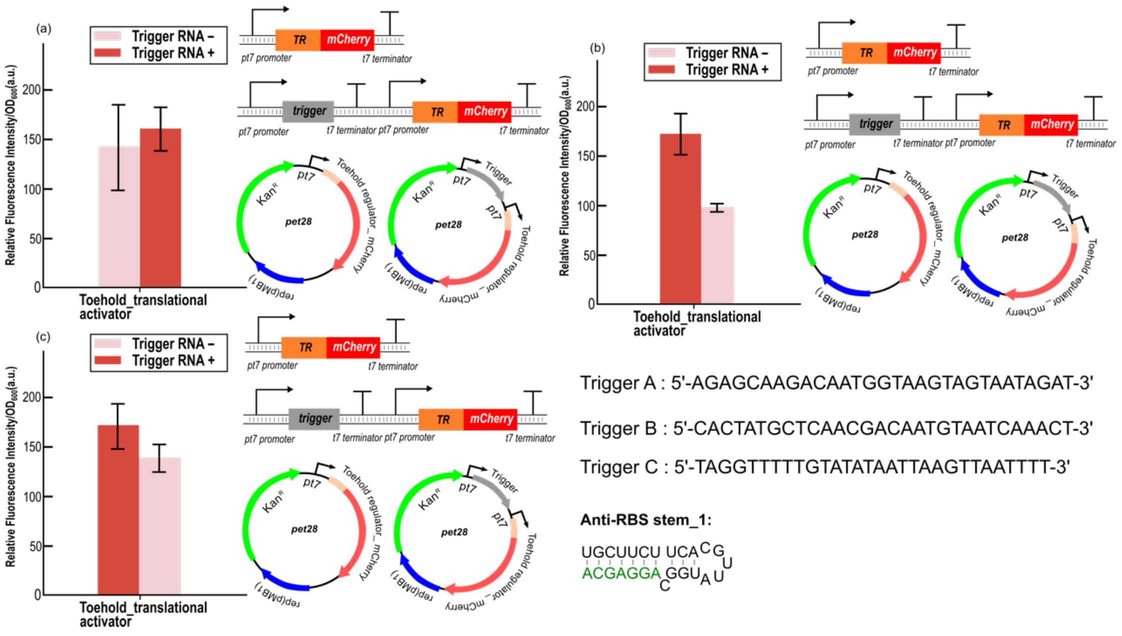

Figure S15 Relative fluorescence intensities of mCherry under the control of toehold-translational activator with non-cognate trigger RNAs. a – c show *in vivo* mCherry expression levels (fluorescence/OD600 end level) under the control of translational toehold activator stem_1 in the absence and presence of the non-cognate triggers A, B, and C, respectively. Expression is measured in *E. coli*, BL21 DE3 with induction of the T7 RNA polymerase by 1mM IPTG. Error bars represent the standard deviation over three biological replicates.

Switching behavior of a translational activator transcribed from a constitutive promoter

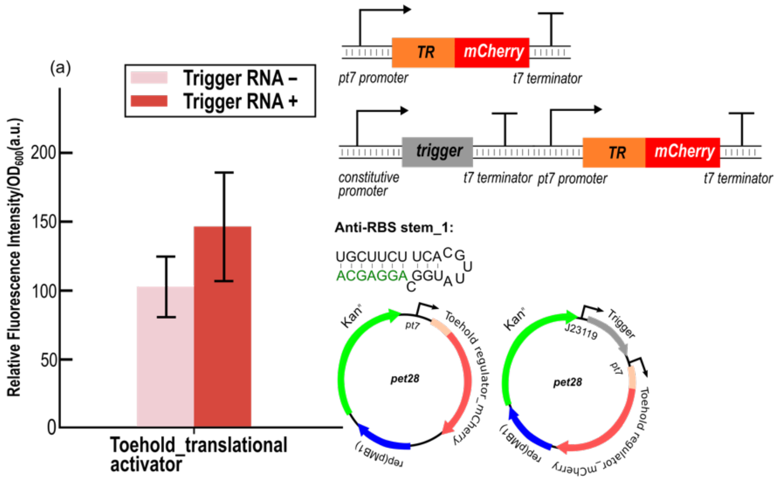

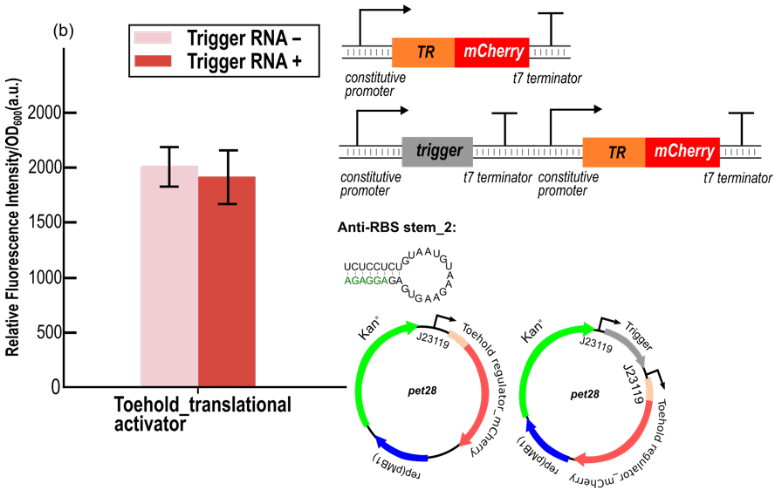

Figure S16 Relative fluorescence intensities of mCherry under the control of toehold-translational activator with constitutive promoter in *E. coli*, BL21 DE3. a, *in vivo* mCherry expression levels (fluorescence/OD600 end level) under the control of translational toehold activator stem_1 in the absence and presence of trigger RNA. Toehold-translational activator is transcribed from a constitutive promoter (BBa_J23119), while trigger RNA is under the control of a pt7 promoter. b, *in vivo* mCherry expression levels (fluorescence/OD600 end level) under the control of translational toehold activator stem_1 in the absence and presence of trigger RNA. Here, both the translational activator and the trigger RNA are controlled transcribed from a constitutive promoter (BBa_J23119). Expression is measured in *E. coli*, BL21 DE3 with induction of the T7 RNA polymerase by 1mM IPTG. Error bars represent the standard deviation obtained from three biological replicates.

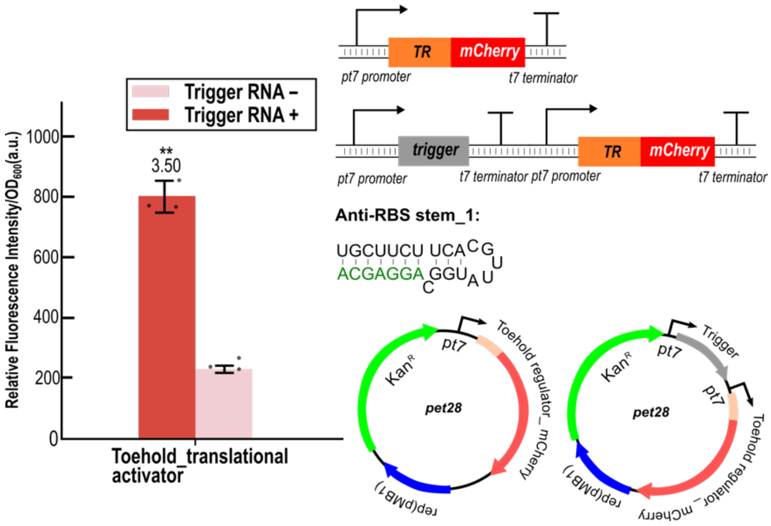

Figure S17. Test of the switch in LB medium. The bar plot shows the relative fluorescence intensities of mCherry (fluorescence/OD600 end level) under the control of toehold-translational activator stem in *E. coli*, BL21 DE3 cultured in LB medium, in the absence and presence of trigger RNA. T7 RNA polymerase is induced by the addition 1mM IPTG. Error bars represent the standard deviation of three biological replicates.

Addition of an hfq binding site to the trigger RNA

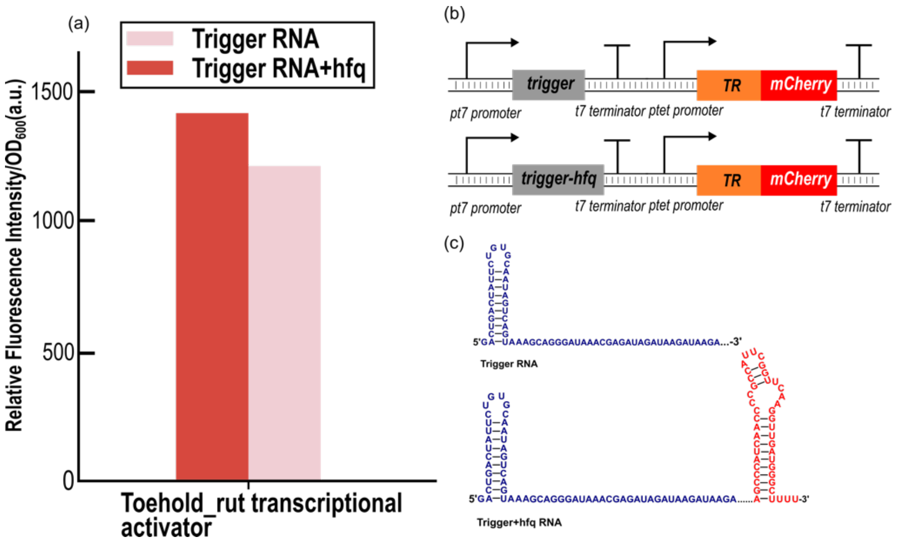

Figure S18. Effect of the RNA chaperone Hfq: Relative fluorescence intensities of mCherry under the control of toehold-anti-rut transcriptional activator in *E. coli*, BL21 DE3 cultured in M9 medium. a, *in vivo* mCherry expression levels (fluorescence/OD600 end level, excitation/emission: 570/620 nm) under the control of transcriptional toehold activator stem_1 in the presence of a standard trigger RNA and a trigger RNA with an hfq hairpin that is recognized by Hfq. Hfq is expected to promote RNA-RNA interactions in E.coli, but only shows a minor effect in this experiment. b, Structure of the gene templats: toehold-activators were controlled by a *ptet* promoter and trigger RNAs were under the control of a pt7 promoter. c, Prediction of mRNA secondary structures of the trigger RNA and trigger RNA with hfq hairpin (red) using NUPACK. Expression is measured in *E. coli*, BL21 DE3 with induction of the T7 RNA polymerase by 1mM IPTG.

A gate implementing IMPLY logic

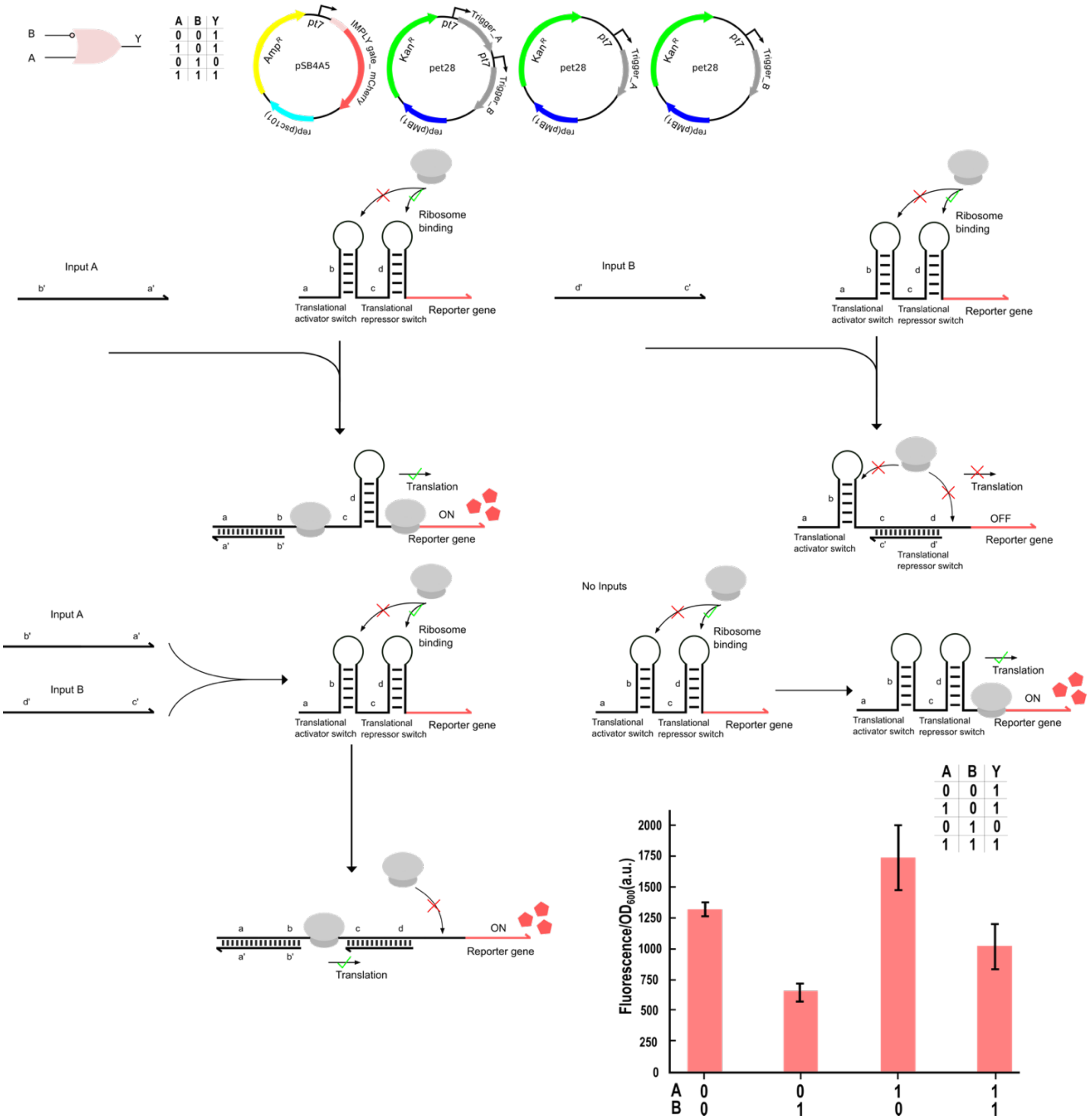

Figure S19. Schematic of a logic gate implementing an IMPLY logic function (B IMPLY A = (NOT B OR A)) by combining a translational activator and inhibitor. As indicated, the IMPLY gate RNA with a downstream mCherry sequence is under the control of a *pt7* promoter on a pSB4A5 low copy number plasmid, whereas trigger RNAs are transcribed from a *pt7* promoter on a pet28 high copy number plasmid with different trigger combinations. The IMPLY gate is comprised of a toehold-translational activator in series with a toehold-translation repressor. In the absence of trigger RNAs, the RBS of the translation activator is sequestered by the anti-RBS sequence, while the RBS of translation repressor is available for ribosome binding, resulting in translation of the downstream reporter gene. In the presence of trigger A alone, translation is activated, in the presence of trigger B alone, translation is inhibited as indicated in the schemes. When both triggers are present, both toehold hairpins are opened. Ribosomes initiating at the RBS exposed by the translation activator can move through the downstream duplex (5'-cd-3'/5'-d'c'-3') and translate the reporter gene. The normalized fluorescence end levels show that the gate behaves as an IMPLY gate, in principle, but there is a relatively strong leak in the presence of trigger B, which is probably the result of the strong individual leak of the translational repressor.
